# Supplementary material for: Preclinical characterization and target validation of the antimalarial pantothenamide MMV693183
Source: Nat Commun. 2022 Apr 20;13:2158. doi: 10.1038/s41467-022-29688-5 (PMC9021288; doi:10.1038/s41467-022-29688-5)
Supplement: Supplementary file 1 — Supplementary information [file 41467_2022_29688_MOESM1_ESM.pdf]

Supplementary Information

Preclinical characterization and target validation of the antimalarial pantothenamide MMV693183

Laura E. de Vries[1#], Patrick A.M. Jansen[2], Catalina Barcelo[3], Justin Munro[4], Julie M.J. Verhoef[1], Charisse Flerida A. Pasaje[5], Kelly Rubiano[6], Josefine Striepen[6], Nada Abla[3], Luuk Berning[7], Judith M. Bolscher[7], Claudia Demarta-Gatsi[3], Rob Henderson[7], Tonnie Huijs[7], Karin M.J. Koolen[7], Patrick K. Tumwebaze[8], Tomas Yeo[6], Anna C.C. Aguiar[9], Iñigo Angulo-Barturen[10], Alisje Churchyard[11], Jake Baum[11], Benigno Crespo Fernández[12], Aline Fuchs[3], Francisco-Javier Gamo[12], Rafael V.C. Guido[9], María Belén Jiménez-Diaz[10], Dhelio B. Pereira[13], Rosemary Rochford[14], Camille Roesch[15,16], Laura M. Sanz[12], Graham Trevitt[17], Benoit Witkowski[15,16], Sergio Wittlin[18,19], Roland A. Cooper[20], Philip J. Rosenthal[21], Robert W. Sauerwein[1,7], Joost Schalkwijk[2], Pedro H.H. Hermkens[22], Roger V. Bonnert[3], Brice Campo[3], David A. Fidock[6], Manuel Llinás[4,23], Jacquin C. Niles[5], Taco W.A. Kooij[1*], Koen J. Dechering[7*]

[1] Department of Medical Microbiology, Radboudumc Center for Infectious Diseases, Radboud Institute for Molecular Life Sciences, Radboud University Medical Center, Nijmegen, The Netherlands; [2] Department of Dermatology, Radboud Institute for Molecular Life Sciences, Radboud University Medical Center, Nijmegen, The Netherlands; [3] Medicines for Malaria Venture, Geneva, Switzerland; [4] Department of Chemistry and Huck Center for Malaria Research, The Pennsylvania State University, University Park, United States of America; [5] Department of Biological Engineering, Massachusetts Institute of Technology, Cambridge, United States of America; [6] Department of Microbiology & Immunology, Columbia University Irving Medical Center, New York, United States of America; [7] TropIQ Health Sciences, Nijmegen, The Netherlands ; [8] Infectious Diseases Research Collaboration, Kampala, Uganda; [9] Sao Carlos Institute of Physics, University of São Paulo, São Carlos, São Paulo, Brazil, São Carlos-SP, Brazil; [10] The Art of Discovery, Derio, Spain; [11] Department of Life Sciences, Imperial College London, South Kensington, London, United Kingdom; [12] Global Health, GlaxoSmithKline, Tres Cantos, Madrid, Spain; [13] Research Center for Tropical Medicine of Rondonia, Porto Velho, Brazil; [14] Department of Immunology and Microbiology, University of Colorado Anschutz School of Medicine, Aurora, United States of America; [15]Malaria Molecular Epidemiology Unit, Institut Pasteur du Cambodge, Phnom Penh, Cambodia; [16]Malaria Translational Research Unit, Institut Pasteur, Paris & Institut Pasteur du Cambodge, Phnom Penh, Cambodia; [17] Sygnature Discovery, Nottingham, United Kingdom; [18] Swiss Tropical and Public Health Institute, Basel, Switzerland; [19] University of Basel, Basel, Switzerland; [20] Department of Natural Sciences and Mathematics, Dominican University of California, San Rafael, USA; [21] Department of Medicine, University of California, San

Francisco, USA; [22] Hermkens Pharma Consultancy, Oss, The Netherlands ; [23] Center for Malaria Therapeutics and Antimicrobial Resistance, Division of Infectious Diseases, Department of Medicine, Columbia University Irving Medical Center, New York, United States of America; [24] Department of Biochemistry & Molecular Biology, The Pennsylvania State University, University Park, United States of America; # Current address: Department of Immunology and Infectious Diseases, Harvard T.H. Chan School of Public Health, Boston, USA; *Contributed equally, Corresponding authors (email: taco.kooij@radboudumc.nl; k.dechering@tropiq.nl)).

Supplementary Methods

Synthesis of PanAms

General Procedure for Amidation-

General Procedure A: To a stirred solution of Amine (1) (1 eqv) and Acid (2) (1.2 eqv) in THF (5 ml/mmol) were added HATU (1.5 eqv) and Et3N (5 eqv) at 0°C. Reaction mixture was stirred at room temperature for 16h. Reaction mixture was quenched with satd. NaHCO3 solution and extracted with EtOAc. The organic layer was washed with water, brine, dried over Na2SO4 and concentrated under reduced pressure. Crude was purified over prep TLC plate (MeOH-DCM) to afford desired amide (3).

General Procedure for acetonide deprotection-

General Procedure B: To a stirred solution of amide (3) (1 eqv) in CH3CN (5 ml/mmol) was treated with Bi(III)Cl3 (0.1 eqv) and H2O (0.04 ml/mmol). The reaction mixture was stirred at room temperature for 16h. Reaction mass was concentrated under reduced pressure and purified over prep TLC (MeOH in DCM) to afford Final compound.

MMV693183

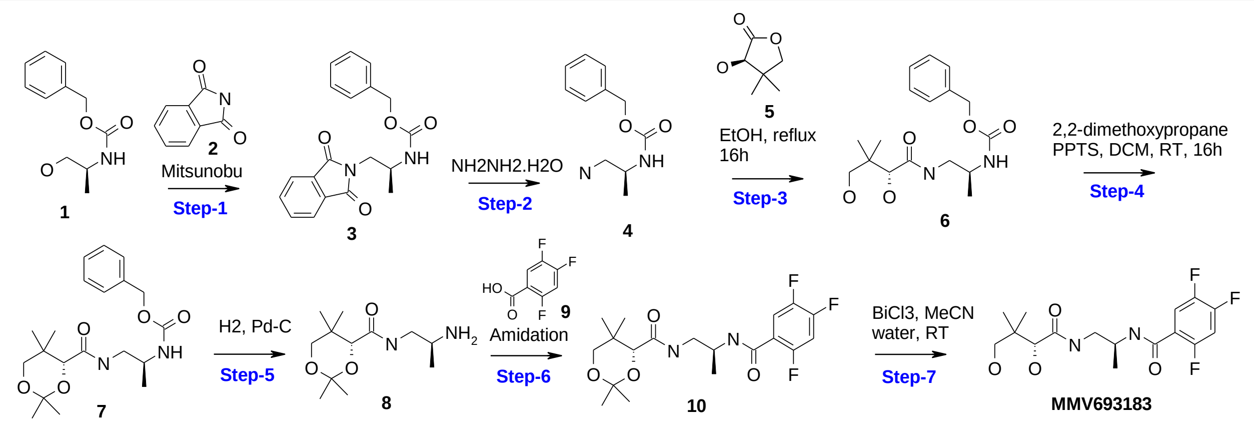

Synthesis of [(S)-2-(1,3-Dioxo-1,3-dihydro-isoindol-2-yl)-1-methyl-ethyl]-carbamic acid benzyl ester (3):

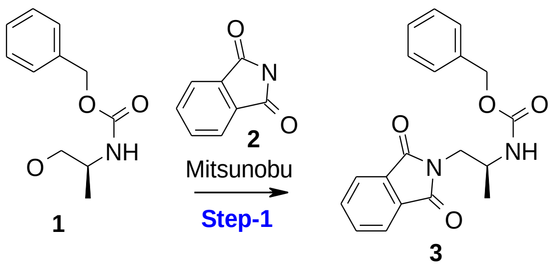

Procedure: To a stirred solution of ((S)-2-Hydroxy-1-methyl-ethyl)-carbamic acid benzyl ester (1) (50 g, 238.9 mmol) in dry THF (1500 mL) were added Phthalimide (2) (38.6 g, 262.8 mmol) and PPh3 (68.9 g, 262.8 mmol). Then DEAD (41.2 mL, 262.8 mmol) was added drop wise at 0^0C. Reaction was stirred at RT for 16h. The reaction mixture was then concentrated and the residue was purified by column chromatography (silica gel, 100-200 mesh) using 20%EtOAc in hexane to afford [(S)-2-(1,3-Dioxo-1,3-dihydro-isoindol-2-yl)-1-methyl-ethyl]-carbamic acid benzyl ester (3) (53 g, 65.5%) as off white solid.

Synthesis of ((S)-2-Amino-1-methyl-ethyl)-carbamic acid benzyl ester (4):

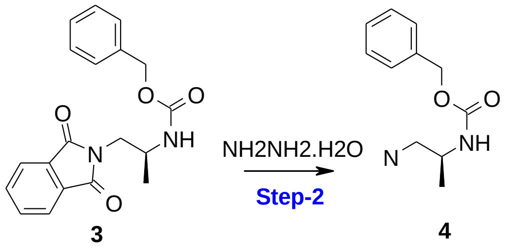

Procedure: To a stirred solution of [(S)-2-(1,3-Dioxo-1,3-dihydro-isoindol-2-yl)-1-methyl-ethyl]-carbamic acid benzyl ester (3) (53 g, 47.7 mmol) in EtOH (450 mL) was added hydrazine hydrate (116.8 mL, 2406.4 mmol). Reaction mass was heated at 50°C for 2h. Reaction mass was cooled to RT, filtered and concentrated under reduced pressure. The resulting residue was again suspended in Et2O and filtered. The combined filtrates were concentrated under reduced pressure to afford ((S)-2-Amino-1-methyl-ethyl)-carbamic acid benzyl ester (4) (30 g, 96%) as colorless sticky gum.

Synthesis of [(S)-2-((R)-2,4-Dihydroxy-3,3-dimethyl-butyrylamino)-1-methyl-ethyl]-carbamic acid benzyl ester (6):

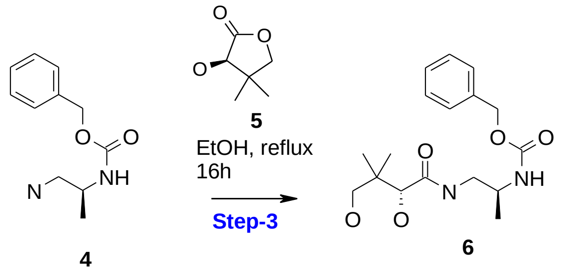

Procedure: To a stirred solution of ((S)-2-Amino-1-methyl-ethyl)-carbamic acid benzyl ester (4) (26 g, 124.8 mmol) in EtOH (150 mL) were added D-(-)-Pantolactone (5) (48.7 g, 374.5 mmol) and Et3N (60.9 mL, 436.9 mmol). Reaction mixture was refluxed for 16h. Reaction mixture was concentrated under reduced pressure to get crude mass which was purified by column chromatography (silica gel, 100-200 mesh) using 3% MeOH in DCM to afford [(S)-2-((R)-2,4-Dihydroxy-3,3-dimethyl-butyrylamino)-1-methyl-ethyl]-carbamic acid benzyl ester (6) (31.2 g, 74%) as colorless sticky gum.

Synthesis of {(S)-1-Methyl-2-[((R)-2,2,5,5-tetramethyl-[1,3]dioxane-4-carbonyl)-amino]-ethyl}-carbamic acid benzyl ester (7):

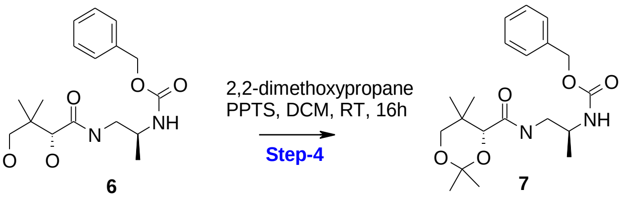

Procedure: To a stirred solution of [(S)-2-((R)-2,4-Dihydroxy-3,3-dimethyl-butyrylamino)-1-methyl-ethyl]-carbamic acid benzyl ester (6) (31.2 g, 92.2 mmol) in DCM (270 mL) were added pyrdinium-p-toluenesulfonate (9.2 g , 36.2 mmol) and 2,2-dimethoxypropane (113.3 g, 922 mmol). Reaction mixture was stirred at RT for 16h. Solvent was removed under reduced pressure and the crude product was purified by column chromatography (silica gel, 100-200 mesh) using 1% MeOH in DCM to afford {(S)-1-Methyl-2-[((R)-2,2,5,5-tetramethyl-[1,3]dioxane-4-carbonyl)-amino]-ethyl}-carbamic acid benzyl ester (7) (22 g, 63%) as colorless sticky gum.

Synthesis of of (R)-2,2,5,5-Tetramethyl-[1,3]dioxane-4-carboxylic acid ((S)-2-amino-propyl)-amide (8):

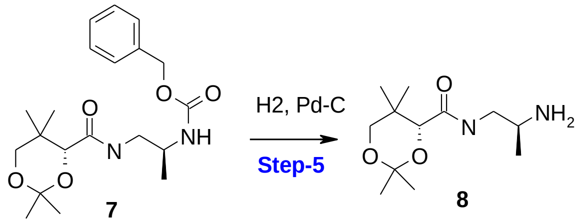

Procedure: To a stirred solution of {(S)-1-Methyl-2-[((R)-2,2,5,5-tetramethyl-[1,3]dioxane-4-carbonyl)-amino]-ethyl}-carbamic acid benzyl ester (7) (22 g, 58.1 mmol) in MeOH (160 mL) was degassed with nitrogen gas for 10 mins then 10% Pd/C (3 g) was added. Reaction mass was stirred at RT under hydrogen atmosphere (balloon pressure) for 3h. Reaction mixture was filtered through celite and the filtrate was concentrated under reduced pressure to afford (R)-2,2,5,5-Tetramethyl-[1,3]dioxane-4-carboxylic acid ((S)-2-amino-propyl)-amide (8) (14 g, 98.5%) as colorless sticky gum.

Synthesis of (R)-2,2,5,5-Tetramethyl-[1,3]dioxane-4-carboxylic acid [(S)-2-(2,4,5-trifluoro-benzoylamino)-propyl]-amide (10):

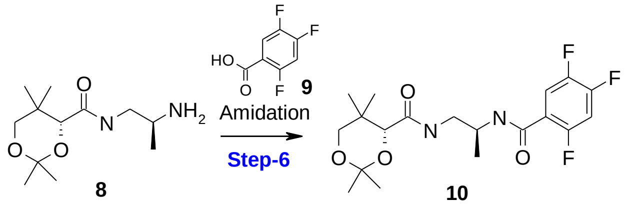

Procedure: Same as general procedure A with (R)-2,2,5,5-Tetramethyl-[1,3]dioxane-4-carboxylic acid ((S)-2-amino-propyl)-amide (8) (5 g, 20.492 mmol) and 2,4,5-Trifluoro-benzoic acid (9) (3.609 g, 20.492 mmol) to afford (R)-2,2,5,5-Tetramethyl-[1,3]dioxane-4-carboxylic acid [(S)-2-(2,4,5-trifluoro-benzoylamino)-propyl]-amide (10) as gum in 60.63% yield, 5 g.

Synthesis of compound MMV693183:

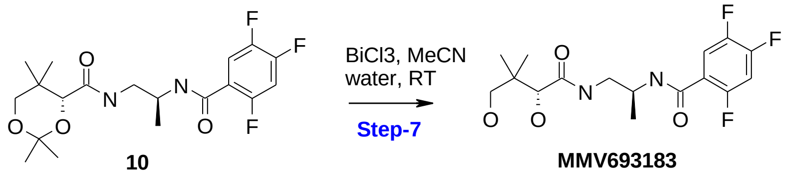

Procedure: Same as general procedure B with (R)-2,2,5,5-Tetramethyl-[1,3]dioxane-4-carboxylic acid [(S)-2-(2,4,5-trifluoro-benzoylamino)-propyl]-amide (10) (5 g, 12.425 mmol) to afford MMV693183 as light yellow sticky solid in 77.74% yield, 3.5 g.

MMV693182

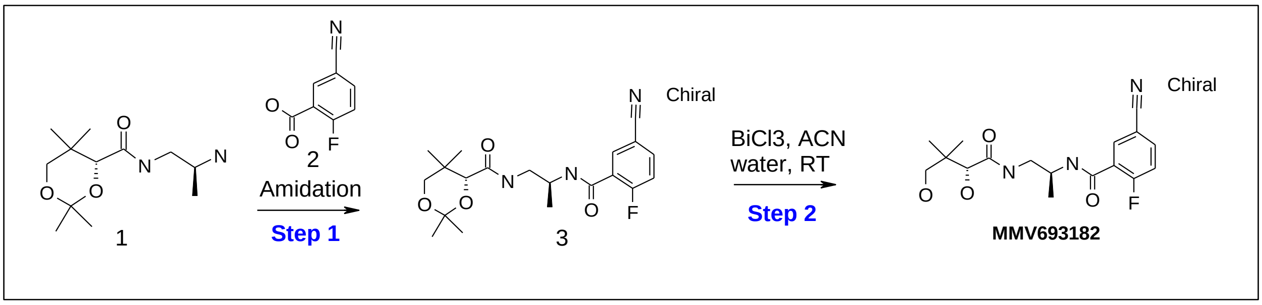

Synthesis of (R)-2,2,5,5-Tetramethyl-[1,3]dioxane-4-carboxylic acid [(S)-2-(5-cyano-2-fluoro-benzoylamino)-propyl]-amide (3):

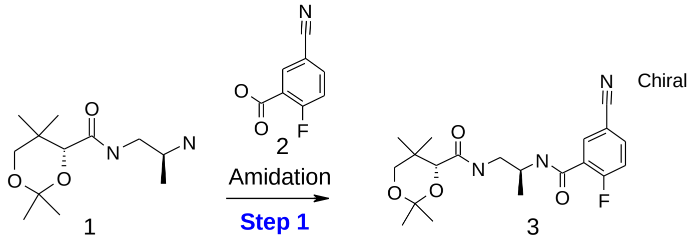

Procedure: Same as general procedure A with (R)-2,2,5,5-Tetramethyl-[1,3]dioxane-4-carboxylic acid ((S)-2-amino-propyl)-amide (1) (4 g, 16.393 mmol) and 5-Cyano-2-fluoro-benzoic acid (2) (2.707 g, 16.393 mmol) to afford (R)-2,2,5,5-Tetramethyl-[1,3]dioxane-4-carboxylic acid [(S)-2-(5-cyano-2-fluoro-benzoylamino)-propyl]-amide (3) as gum in 56.57 % yield, 3.63 g.

Synthesis of compound MMV693182:

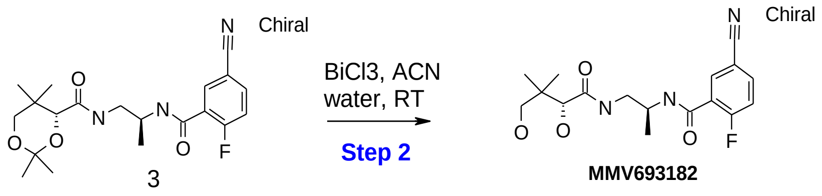

Procedure: Same as general procedure B with (R)-2,2,5,5-Tetramethyl-[1,3]dioxane-4-carboxylic acid [(S)-2-(5-cyano-2-fluoro-benzoylamino)-propyl]-amide (3) (3.63 g, 9.273 mmol) to afford MMV693182 as off white sticky solid in 73.66% yield, 2.4 g.

MMV884962

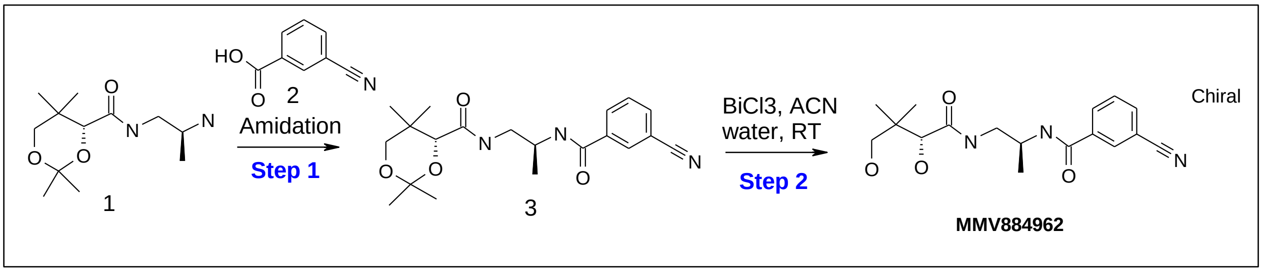

Synthesis of (R)-2,2,5,5-Tetramethyl-[1,3]dioxane-4-carboxylic acid [(S)-2-(3-cyano-benzoylamino)-propyl]-amide (3):

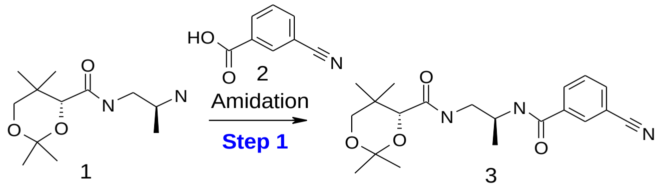

Procedure: Same as general procedure A with (R)-2,2,5,5-Tetramethyl-[1,3]dioxane-4-carboxylic acid ((S)-2-amino-propyl)-amide (1) (5 g, 20.463 mmol) and 3-Cyano-benzoic acid (2) (3.011 g, 20.463 mmol) to afford (R)-2,2,5,5-Tetramethyl-[1,3]dioxane-4-carboxylic acid [(S)-2-(3-cyano-benzoylamino)-propyl]-amide (3) as gum in 50.32% yield, 4.2 g.

Synthesis of compound MMV884962:

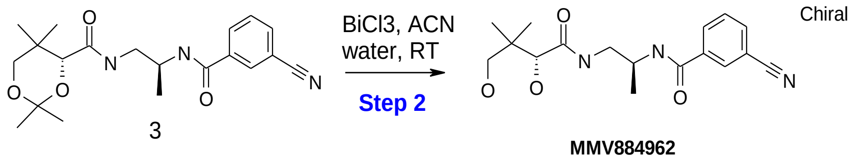

Procedure: Same as general procedure B with (R)-2,2,5,5-Tetramethyl-[1,3]dioxane-4-carboxylic acid [(S)-2-(3-cyano-benzoylamino)-propyl]-amide (3) (4.2 g, 11.246 mmol) to afford MMV884962 as off white solid in 82.68% yield, 3.1 g.

MMV1542001

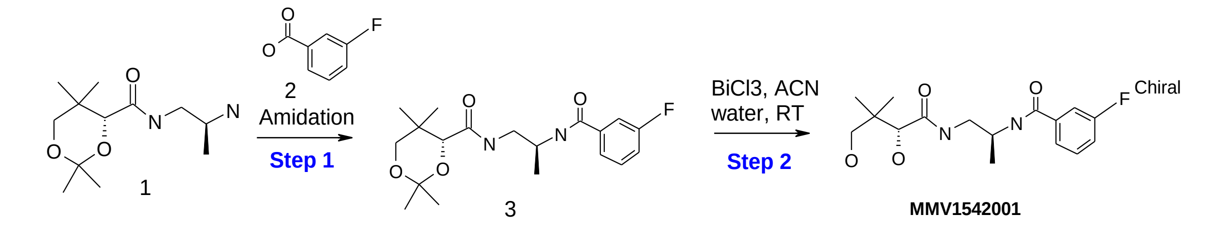

Synthesis of (R)-2,2,5,5-Tetramethyl-[1,3]dioxane-4-carboxylic acid [(S)-2-(3-fluoro-benzoylamino)-propyl]-amide (3):

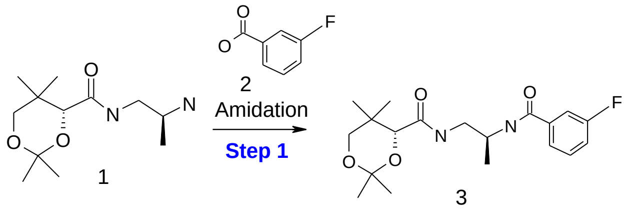

Procedure: Same as general procedure A with (R)-2,2,5,5-Tetramethyl-[1,3]dioxane-4-carboxylic acid ((S)-2-amino-propyl)-amide (1) (3 g, 12.295 mmol) and 3-Fluoro-benzoic acid (2) (1.723 g, 12.295 mmol) to afford (R)-2,2,5,5-Tetramethyl-[1,3]dioxane-4-carboxylic acid [(S)-2-(3-fluoro-benzoylamino)-propyl]-amide (3) as gum in 77.69% yield, 3.5 g.

Synthesis of compound MMV1542001:

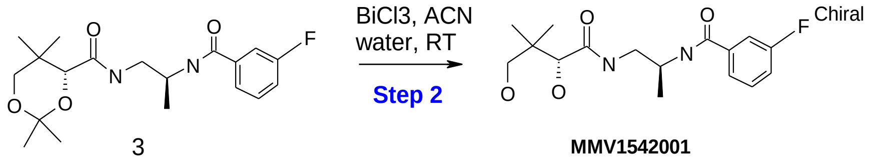

Procedure: Same as general procedure B with (R)-2,2,5,5-Tetramethyl-[1,3]dioxane-4-carboxylic acid [(S)-2-(3-fluoro-benzoylamino)-propyl]-amide (3) (3.5 g, 9.552 mmol) to afford MMV1542001 as off white solid in 63.56% yield, 2.2 g.

MMV976394

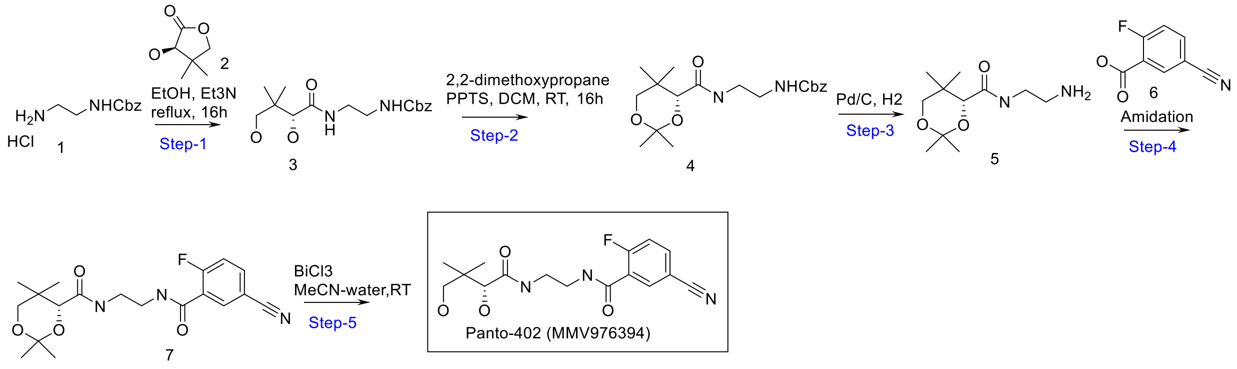

Synthesis of benzyl (R)-(2-(2,4-dihydroxy-3,3-dimethylbutanamido)ethyl)carbamate (3):

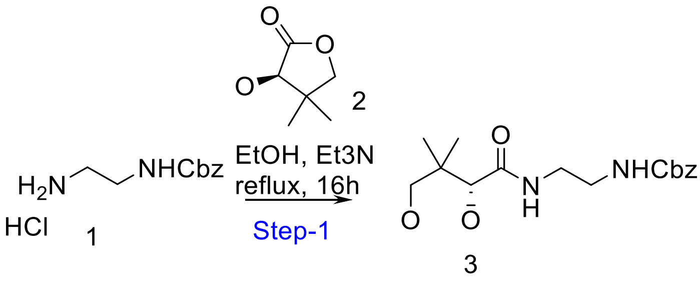

Procedure: To a stirred solution of **benzyl (2-aminoethyl)carbamate hydrochloride (1)** (6 g, 25.992 mmol) in EtOH (75 ml) were added **(R)-3-hydroxy-4,4-dimethyldihydrofuran-2(3H)-one (2)** (10.15 g, 77.976 mmol) and triethyl amine (18.11 ml, 129.96 mmol). The reaction mixture was heated to a gentle reflux for 16 h. The solvent was removed under reduced pressure and the crude product was purified

by column chromatography (silica gel, 100-200 mesh) using 70%EtOAc in hexane to afford **benzyl (R)-(2-(2,4-dihydroxy-3,3-dimethylbutanamido)ethyl)carbamate (3)** (8 gm, 94.89 %) as a yellow oil .

Synthesis of benzyl (R)-(2-(2,2,5,5-tetramethyl-1,3-dioxane-4-carboxamido)ethyl)carbamate (4):

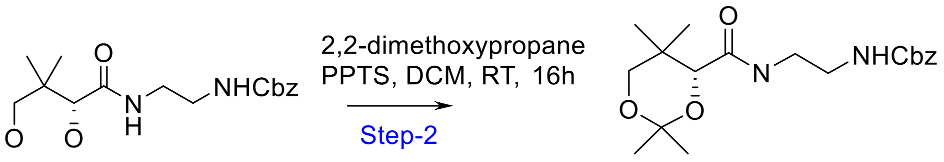

3 4

Procedure: To a stirred solution of **benzyl (R)-(2-(2,4-dihydroxy-3,3-dimethylbutanamido)ethyl)carbamate (3)** (10 g, 30.864 mmol) in DCM (80 mL) were added pyrdinium-p-toluenesulfonate (2.87 g, 11.42 mmol) and 2,2-dimethoxypropane (37.82 ml, 308.642 mmol). Reaction mixture was stirred at RT for 16h. Solvent was removed under reduced pressure and the crude product was purified by column chromatography (silica gel, 100-200 mesh) using 50% EtOAc in hexane to afford **benzyl (R)-(2-(2,2,5,5-tetramethyl-1,3-dioxane-4-carboxamido)ethyl)carbamate (4)** (9.4 g, 83.57 %) as colorless sticky gum.

Synthesis of (R)-N-(2-aminoethyl)-2,2,5,5-tetramethyl-1,3-dioxane-4-carboxamide (5):

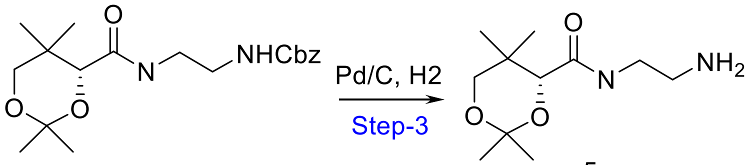

4 5

Procedure: A stirred solution of **benzyl (R)-(2-(2,2,5,5-tetramethyl-1,3-dioxane-4-carboxamido)ethyl)carbamate (4)** (9 g, 25.568 mmol) in MeOH (80 mL) was degassed with nitrogen gas for 15 mins then 10% Pd/C (2 g) was added. Reaction mass was stirred at RT under hydrogen atmosphere (balloon pressure) for 2h. Reaction mixture was filtered through celite and the filtrate was concentrated under reduced pressure to afford **(R)-N-(2-aminoethyl)-2,2,5,5-tetramethyl-1,3-dioxane-4-carboxamide (5)** (5.4 g, 91.7 %) as colorless sticky gum.

Synthesis of (R)-N-(2-(5-cyano-2-fluorobenzamido)ethyl)-2,2,5,5-tetramethyl-1,3-dioxane-4-carboxamide (7):

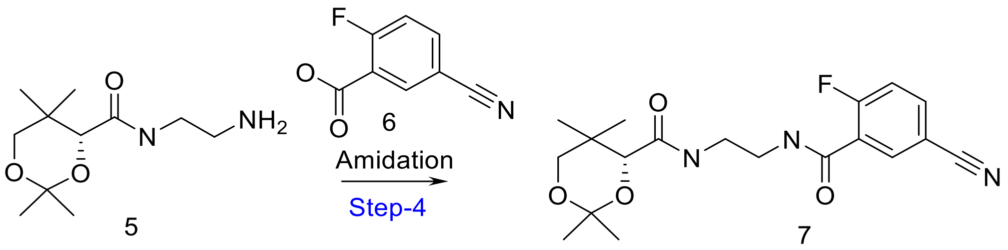

Procedure: To a stirred solution of **(R)-N-(2-aminoethyl)-2,2,5,5-tetramethyl-1,3-dioxane-4-carboxamide (5)** (5.4 g, 23.447 mmol) and **5-cyano-2-fluorobenzoic acid (6)** (3.87 g, 23.447 mmol) in THF (70 ml) were added Et3N (16.34 ml, 117.233 mmol), EDCI (6.72 g, 35.17 mmol) and HOBT (4.75 g, 35.17 mmol) at 0°C. Reaction mixture was stirred at room temperature for 16h. Reaction mixture was quenched with satd. NaHCO3 solution and extracted with EtOAc. The organic layer was washed with water, brine, dried over Na2SO4 and concentrated under reduced pressure. Crude was purified by column chromatography (silica gel, 100-200 mesh) using 2% MeOH in DCM to afford **(R)-N-(2-(5-cyano-2-fluorobenzamido)ethyl)-2,2,5,5-tetramethyl-1,3-dioxane-4-carboxamide (7)** (6.7 g, 75.71%) as colorless sticky gum.

Synthesis of (R)-5-cyano-N-(2-(2,4-dihydroxy-3,3-dimethylbutanamido)ethyl)-2-fluorobenzamide (MMV976394):

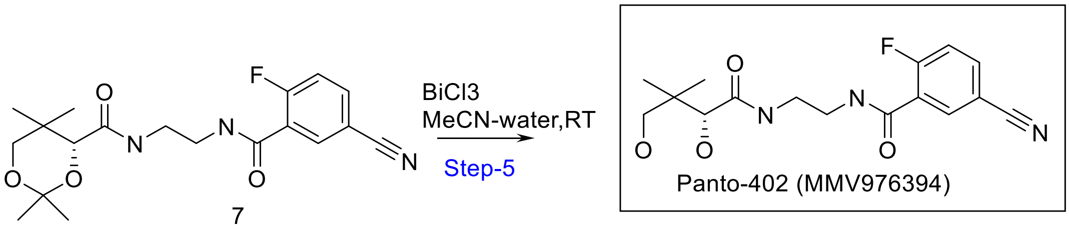

Procedure: To a stirred solution of **(R)-N-(2-(5-cyano-2-fluorobenzamido)ethyl)-2,2,5,5-tetramethyl-1,3-dioxane-4-carboxamide (7)** (6.7 g, 17.752 mmol) in CH3CN (50 ml) was treated with Bi(III)Cl3 (1.12 g, 3.55 mmol) and H2O (0.1 ml). The reaction mixture was stirred at room temperature for 5h. Reaction mass was concentrated under reduced pressure and crude was purified by column chromatography (silica gel, 100-200 mesh) using 2% MeOH in DCM to afford **(R)-5-cyano-N-(2-(2,4-dihydroxy-3,3-dimethylbutanamido)ethyl)-2-fluorobenzamide (MMV976394)** (2.8 g, 46.76 %) as off white solid.

^{1}H NMR (400 MHz, DMSO-d_6) δ 8.52 (brs, 1H), 8.10-8.05 (m, 2H), 7.89 (brs, 1H), 7.56 (t, J= 9.12Hz , 1H), 5.38 (d, J = 5.24Hz, 1H), 4.46 (brs, 1H), 3.72 (d, J= 5.24Hz, 1H), 3.32-3.10 (m, 6H), 0.80 (s, 3H), 0.78 (s, 3H).

LCMS (HCOOH:ACN): M+H=338, R_t=1.38 min in 3 mins run.

Pantothenamide_^{1}H NMR and LCMS

MMV689258:

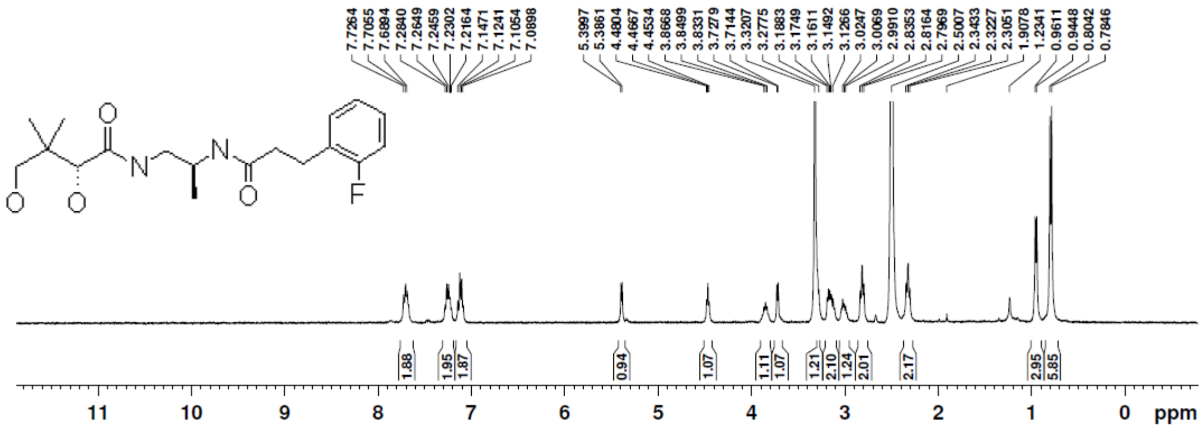

Figure MMV189258 ^{1}H NMR: (400 MHz, DMSO-d_6) δ 0.79 (d, 6 H), 0.95 (d, 3H), 2.32 (t, 2H), 2.81 (t, 2H), 2.99-3.02 (m, 1H), 3.12-3.18 (m, 2H), 3.27-3.32 (m, 1H), 3.72 (d, 1H), 3.83-3.86 (m, 1H), 4.46 (t, 1H, -OH), 5.38 (d, 1H, -OH), 7.08-7.14 (m, 2H), 7.21-7.28 (m, 2H), 7.68-7.72 (m, 2H).

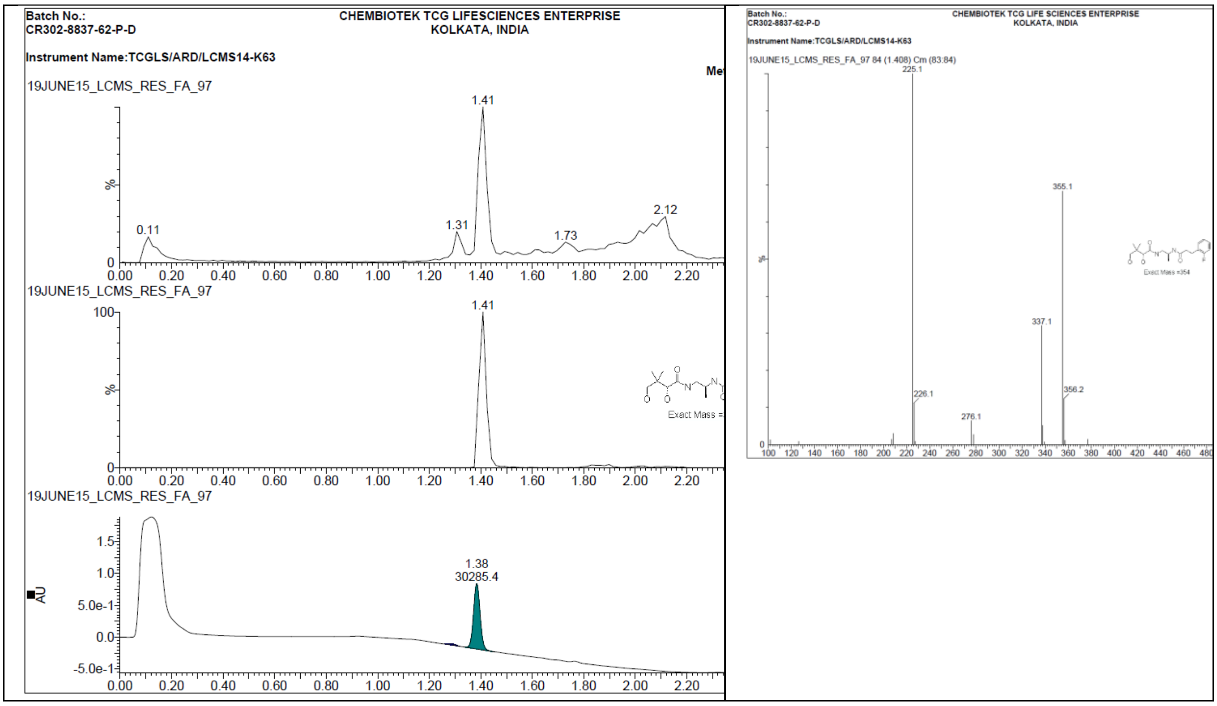

Figure MMV189258 LCMS: (HCOOH:ACN): M+H=355, Rt=1.38 min in 3 mins run

MMV693183:

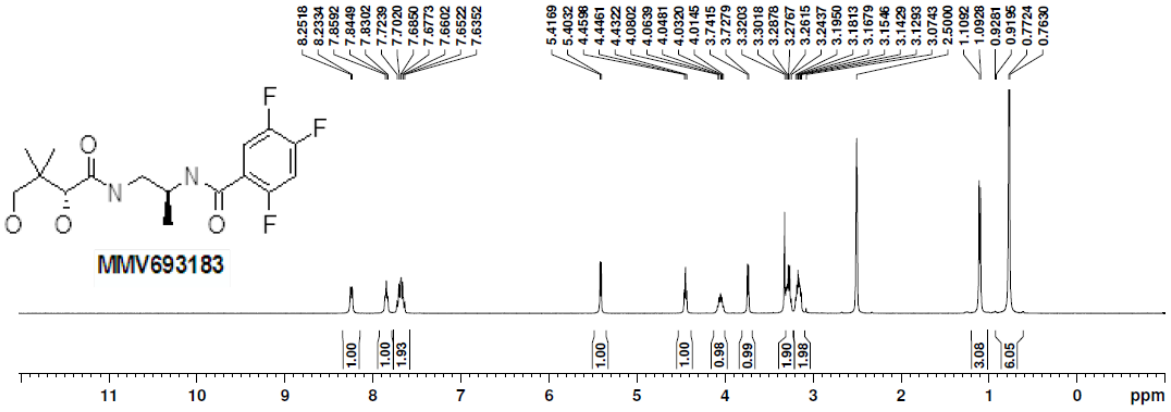

Figure MMV693183 ^{1}H NMR: (400 MHz, DMSO-d_6) δ 8.24 (d, J = 7.56Hz, 1H), 7.84 (t, J = 5.88Hz, 1H), 7.72-7.63 (m, 2H), 5.41 (d, J = 5.48Hz, 1H), 4.44 (t, J = 5.58Hz, 1H), 4.07-4.03 (m, 1H), 3.73 (d, J = 5.48Hz, 1H), 3.32-3.24 (m, 2H), 3.19-3.12 (m, 2H), 1.10 (d, J = 6.64Hz, 3H), 0.77 (s, 3H), 0.76 (s, 3H).

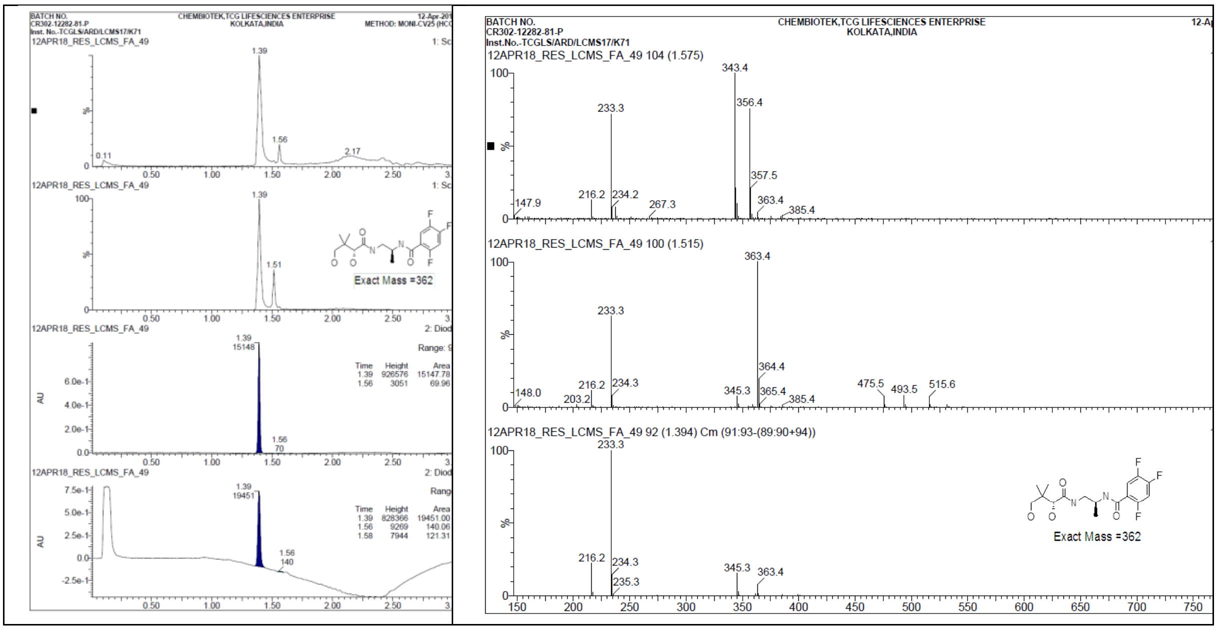

Figure MMV693183 LCMS: (HCOOH:ACN): M+H=363, R_t=1.46 min in 3 mins run.

MMV693182:

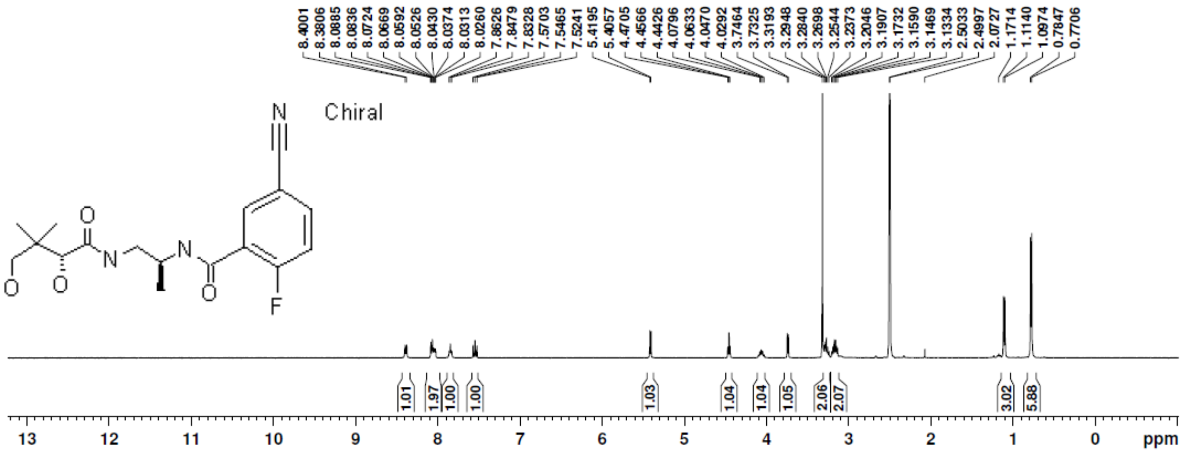

Figure MMV693182 ^{1}H NMR: (400 MHz, DMSO-d_6) δ 8.39 (d, J = 7.8Hz, 1H), 8.08-8.02 (m, 2H), 7.84 (t, J = 5.96Hz, 1H), 7.54 (t, J = 9.24Hz, 1H), 5.41 (d, J = 5.52Hz, 1H), 4.45 (t, J = 5.58Hz, 1H), 4.08-4.02 (m, 1H), 3.74 (d, J = 5.56Hz, 1H), 3.31-3.23 (m, 2H), 3.20 (m, 2H), 1.10 (d, J = 6.64Hz, 3H), 0.78 (s, 3H), 0.77 (s, 3H).

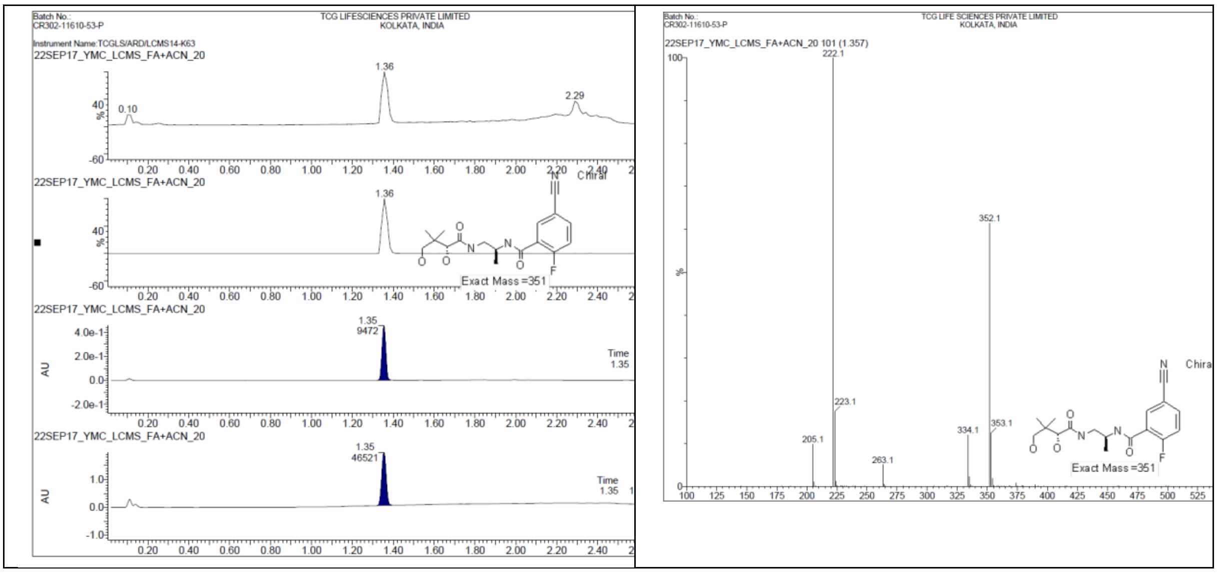

Figure MMV693182 LCMS: (HCOOH:ACN): M+H=352, R_t=1.35 min in 3 mins run.

MMV884962:

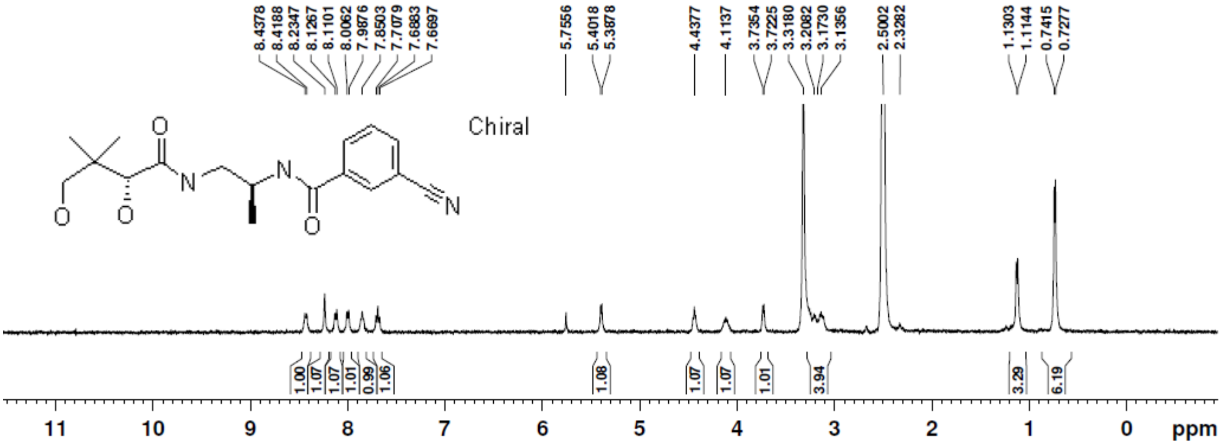

Figure MMV884962 ^{1}H NMR: (400 MHz, DMSO-d_6) δ 8.42 (d, J = 7.6Hz, 1H), 8.23 (s, 1H), 8.12 (d, J = 6.64Hz, 1H), 7.99 (d, J = 7.44Hz, 1H), 7.85 (brs, 1H), 7.68 (t, J = 7.64Hz, 1H), 5.39 (d, J = 5.6Hz, 1H), 4.43 (brs, 1H), 4.13-4.10 (m, 1H), 3.73 (d, J = 5.6 Hz, 1H), 3.31-3.12 (m, 4H), 1.12 (d, J = 6.36Hz, 3H), 0.74 (s, 3H), 0.72 (s, 3H).

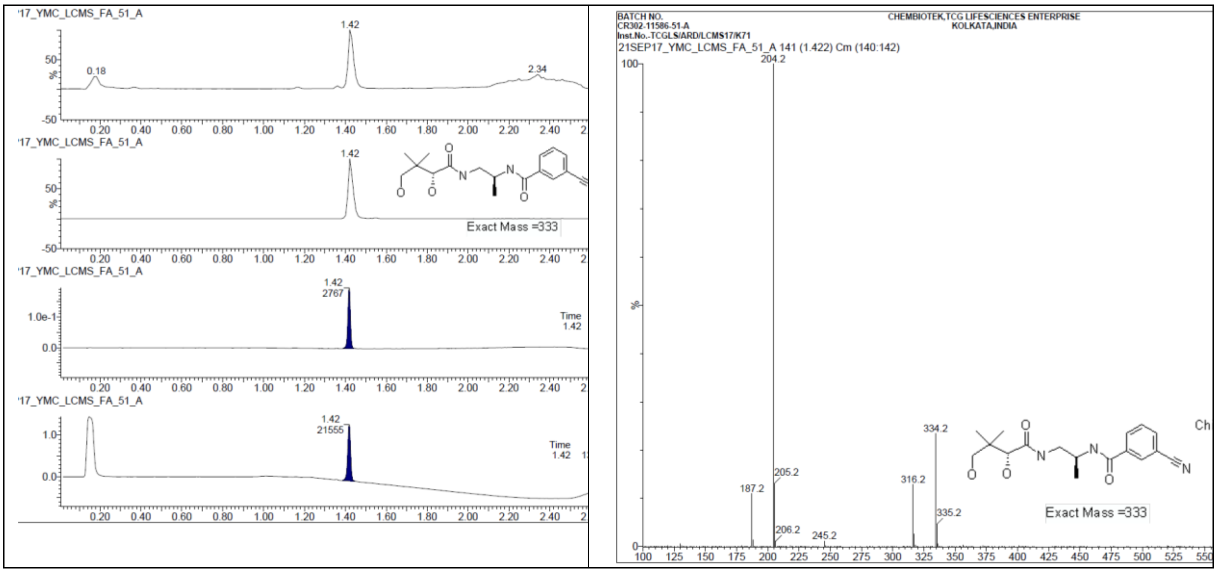

Figure MMV884962 LCMS: (HCOOH:ACN): M+H=334, R_t=1.42 min in 3 mins run.

MMV1542001:

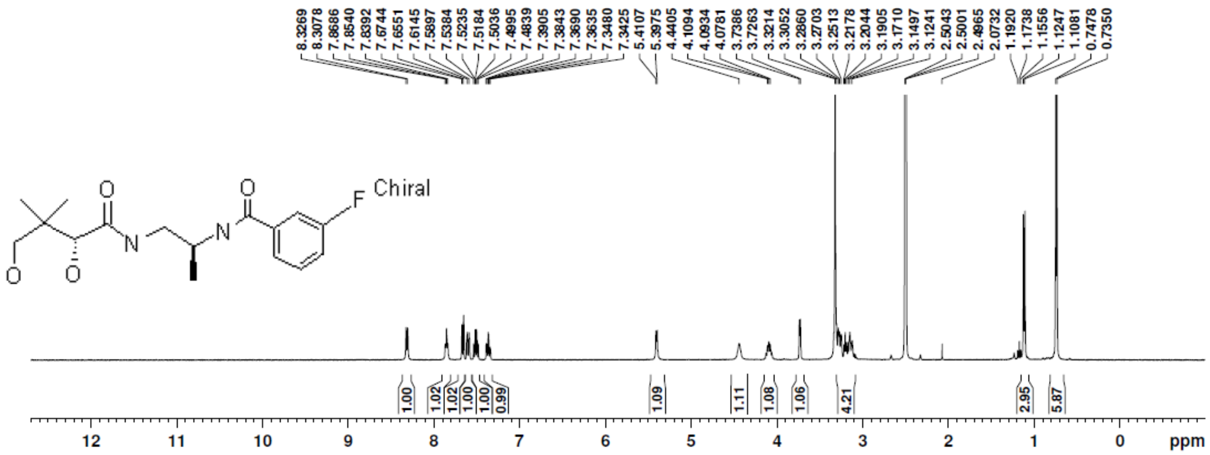

Figure MMV1542001 ^{1}H NMR: (400 MHz, DMSO-d_6) δ 8.31 (d, J = 7.64Hz, 1H), 7.85 (t, J = 5.88Hz, 1H), 7.66 (d, J = 7.72 Hz, 1H), 7.61-7.58 (m, 1H), 7.53-7.48 (m, 1H), 7.39-7.34 (m, 1H), 5.40 (d, J = 5.28Hz, 1H), 4.44 (brs, 1H), 4.12-4.08 (m, 1H), 3.73 (d, J = 5.28Hz, 1H), 3.32-3.12 (m, 4H), 1.11 (d, J = 6.64Hz, 3H), 0.74 (s, 3H), 0.73 (s, 3H).

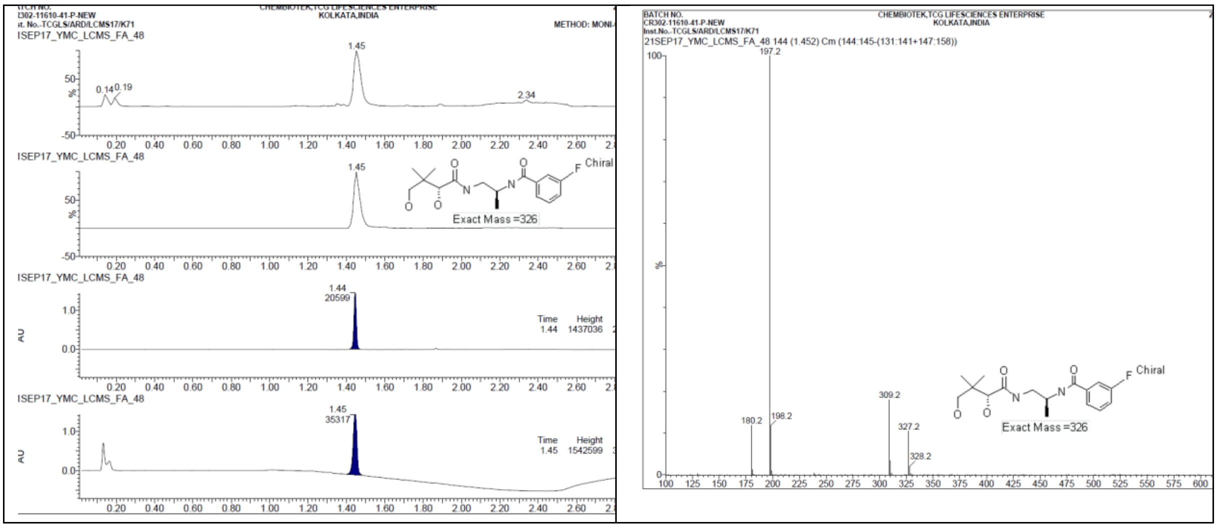

Figure MMV1542001 LCMS: (HCOOH:ACN): M+H=327, R_t=1.45 min in 3 mins run.

MMV976394:

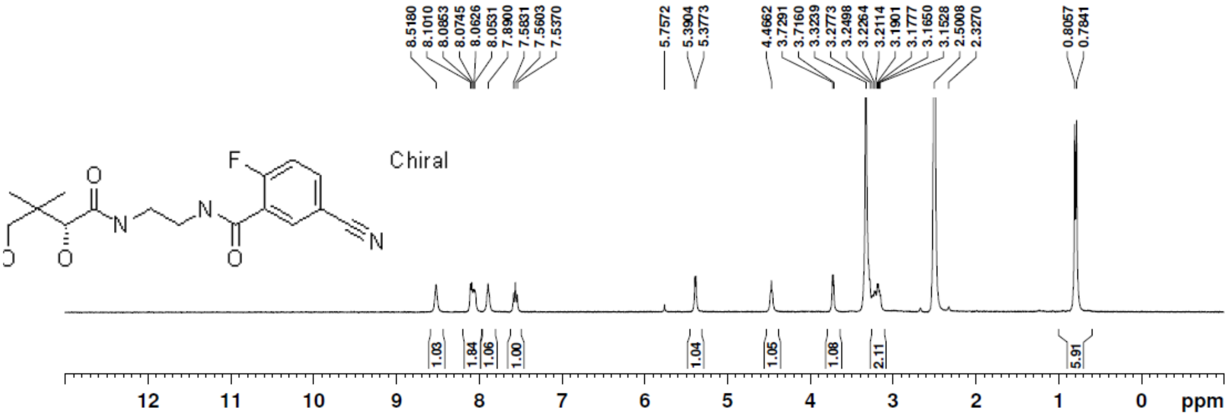

Figure MMV976394 ^{1}H NMR: (400 MHz, DMSO-d_6) δ 8.52 (brs, 1H), 8.10-8.05 (m, 2H), 7.89 (brs, 1H), 7.56 (t, J= 9.12Hz , 1H), 5.38 (d, J = 5.24Hz, 1H), 4.46 (brs, 1H), 3.72 (d, J= 5.24Hz, 1H), 3.32-3.10 (m, 6H), 0.80 (s, 3H), 0.78 (s, 3H).

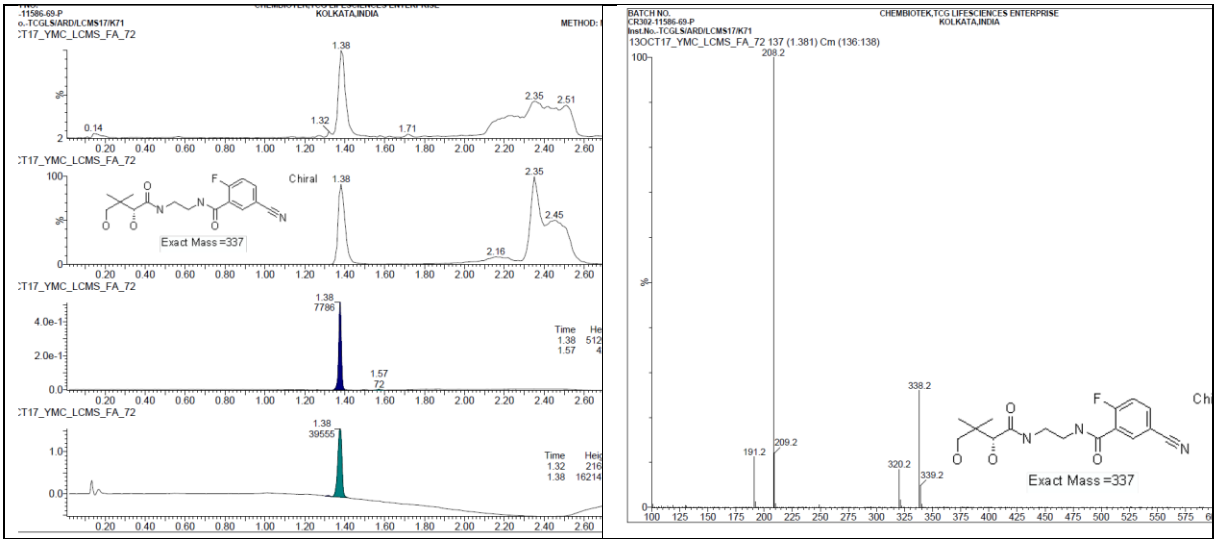

Figure MMV976394 LCMS: (HCOOH:ACN): M+H=338, R_t=1.38 min in 3 mins run.

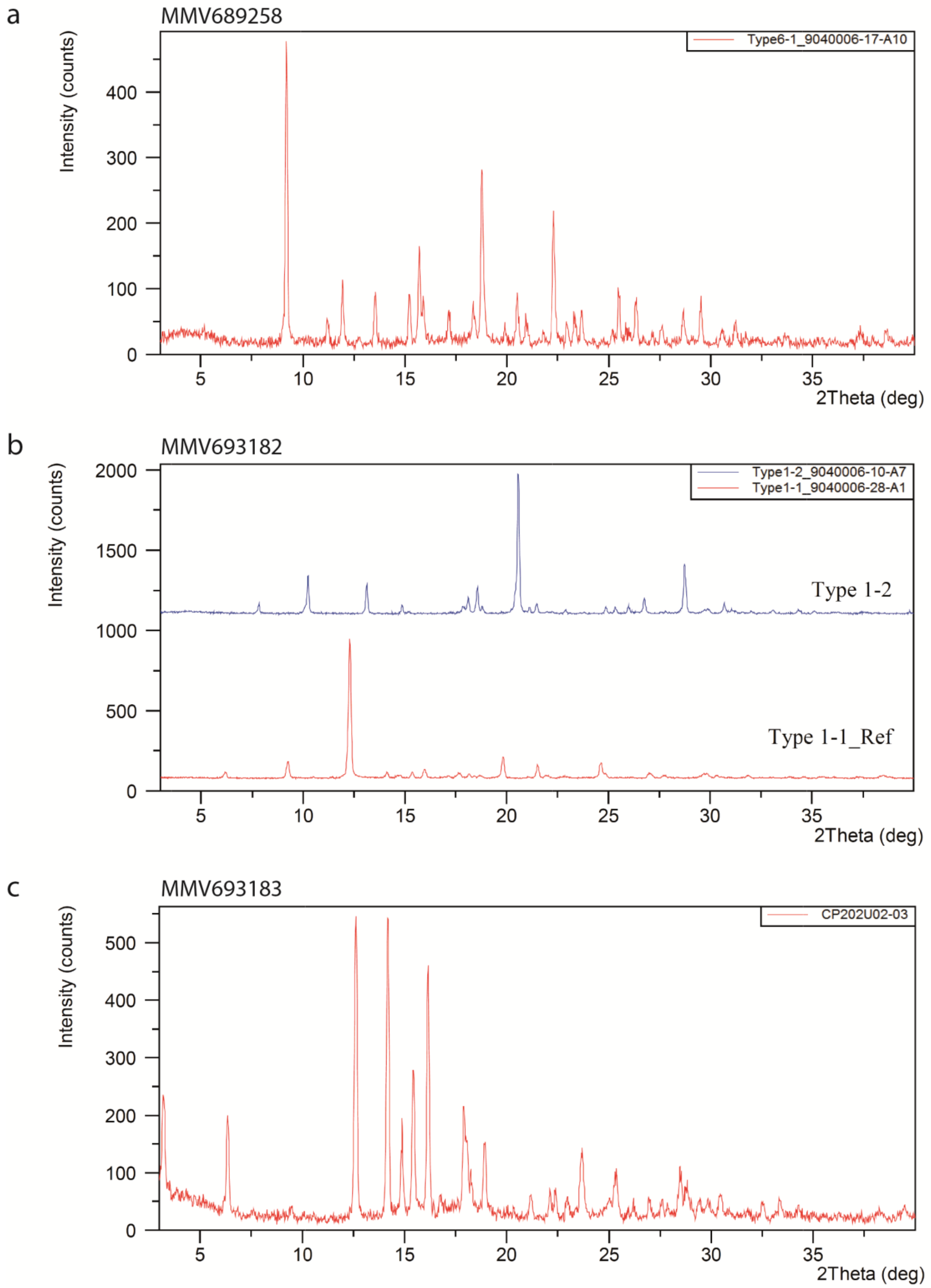

Supplementary Figure 1. XRPD patterns from crystallization screen for crystalline MMV689258 (a), MMV693182 (b) and MMV693183 (c).

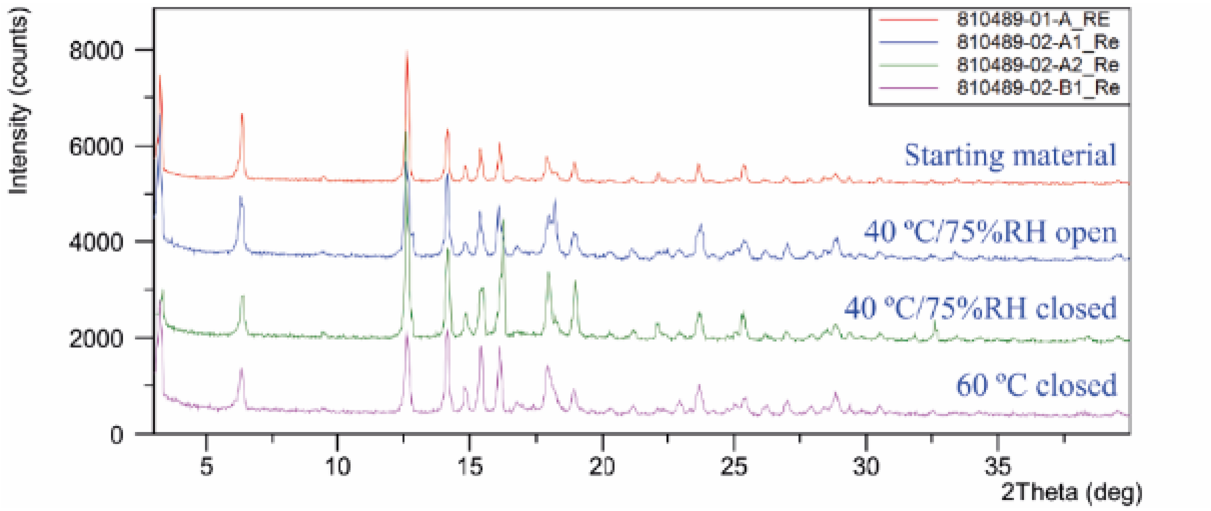

Supplementary Figure 2. XRPD overlay of stability samples. MMV693183 was incubated at different conditions of stress for one month. XRPD patterns show that there is no form-change from the starting material, indicating the stability of this crystalline product.

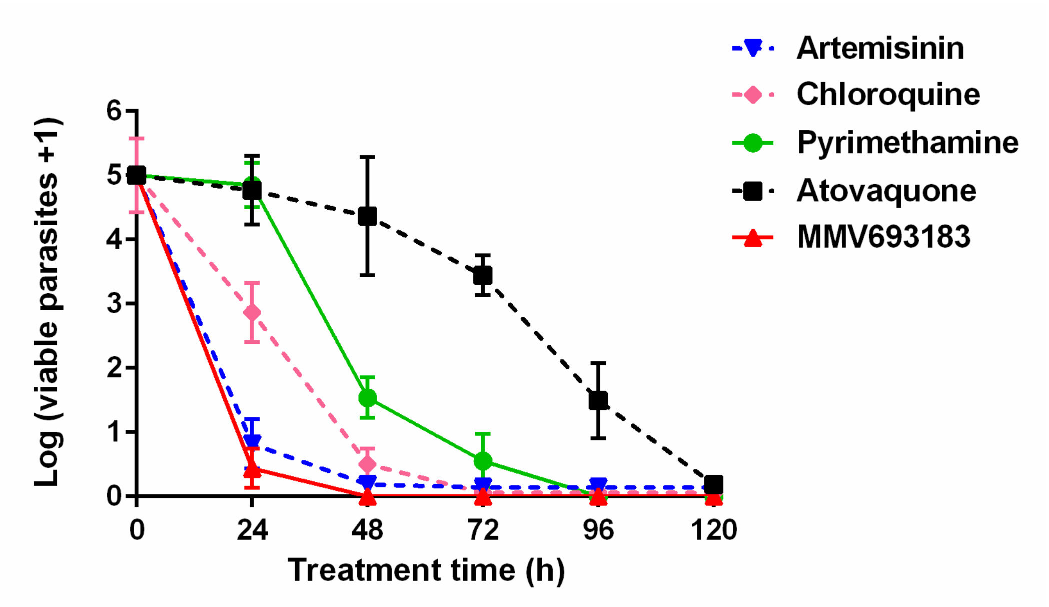

Supplementary Figure 3. *In vitro* antimalarial killing rate. Parasites were treated with 40 nM MMV693183 or 0.94 µM pyrimethamine and the mean number of viable parasites were quantified in one experiment with four technical replicates for MMV693183 and 8 for primaquine (±SD). Artemisinin (0.32 µM), chloroquine (0.39 µM) and atovaquone (0.01 µM) are retrieved from different datasets and shown for comparative purposes. Source data are provided as Source Data file.

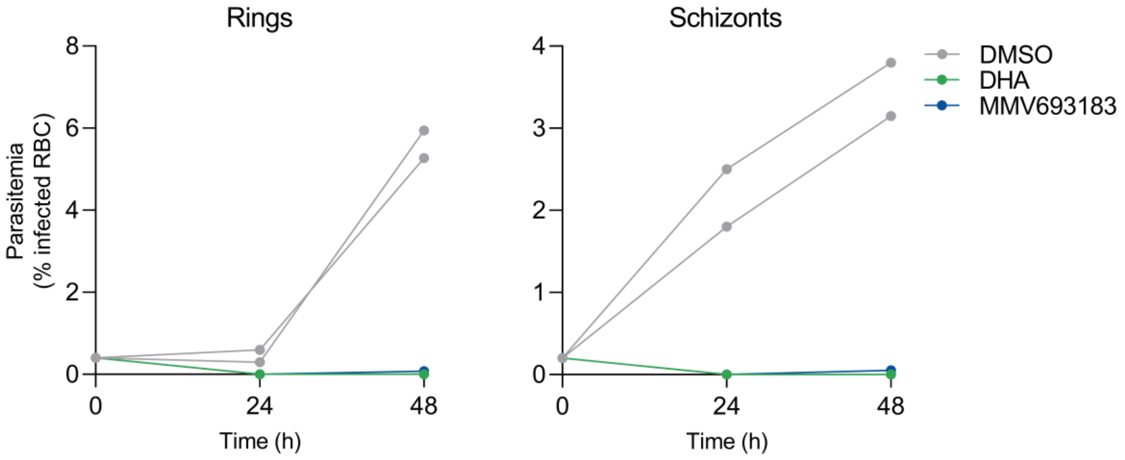

Supplementary Figure 4. MMV693183 activity against asexual blood stages. Synchronized ring (left) or schizont (right) asexual blood stages were exposed to DMSO, 50 nM DHA or 50 nM MMV693183 for 24 hours, followed by washout of the compound. Parasitemia was determined using microscopy in a single experiment with a technical duplicate at 0 h, 24 h and 48 h after exposure, both replicates are shown. Source data are provided as Source Data file.

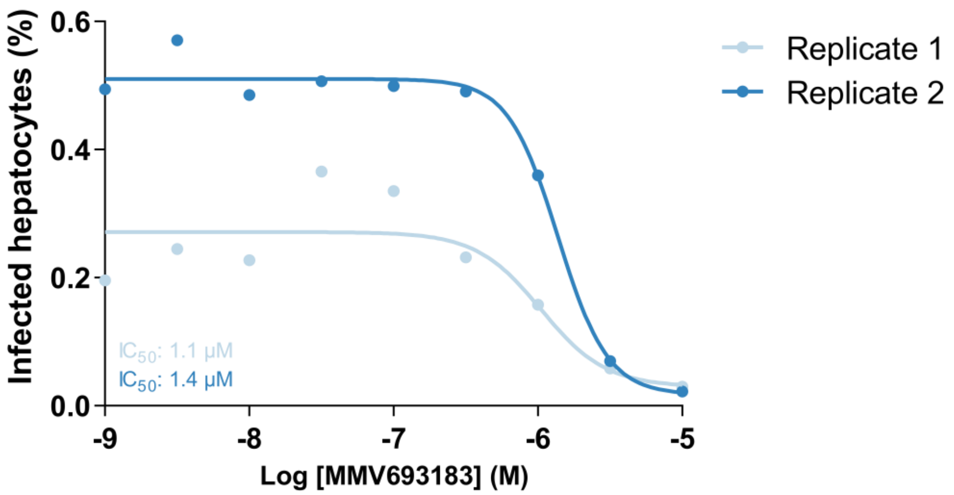

Supplementary Figure 5. MMV693183 is not efficacious against liver stages. Cryopreserved human primary hepatocytes were infected with *P. falciparum* sporozoites and exposed to different concentrations of MMV693183. The number of infected hepatocytes were quantified using microscopy. Data from one experiment with two replicates are shown. While a difference in the baseline number of infected hepatocytes was observed within 1 experiment, both replicates are shown. Reassuringly, both replicates have a similar IC_{50} value. Source data are provided as Source Data file.

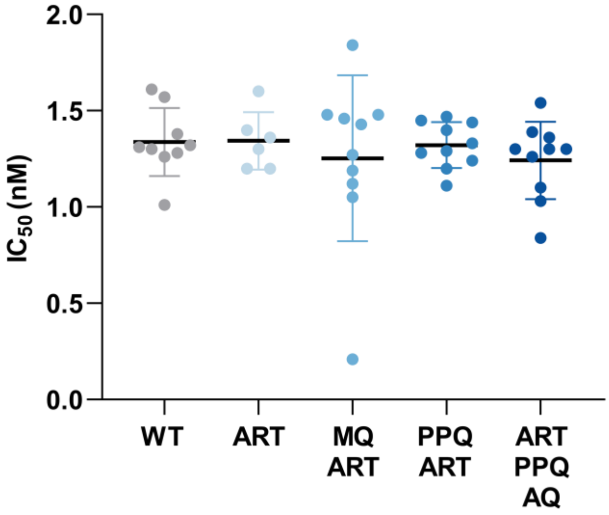

Supplementary Figure 6. **Artemisinin-resistant Cambodian isolates are sensitive to MMV693183**. IC_{50} values of wild-type and artemisinin-resistant parasites from patients in Cambodia grouped according to their resistance pattern. The horizontal bars indicate the median IC_{50} (±SD). WT: parasites sensitive to DHA , MQ, PPQ and mdAQ (N = 9); ART: isolates resistant to DHA, harboring a C580Y or Y493H mutation in *Kelch13* (N = 6); MQ-ART: isolates with pfmdr1 amplification and a C580Y or Y493H mutation in *Kelch13* (N = 10); PPQ-ART: isolates with pfcrt mutations (88, 93, 97 or 145) and/or pfpm2 amplification, and a C580Y mutation in *Kelch13* (N = 10); ART-PPQ-AQ: isolates with a *kelch13* mutation (C580Y or Y493H) and harboring pfpm2 amplification and/or a mutation in pfcrt (93 or 97) (N = 10). ART: artemisinin, PPQ: piperaquine; MQ: mefloquine: AQ: amodiaquine. Source data are provided as Source Data file.

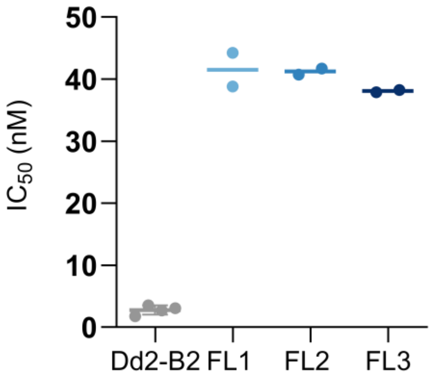

Supplementary Figure 7. Induction of MMV693183 resistant parasites in bulk cultures. 10^9 parasites were exposed to 12 nM MMV693183 ramped up to 27 nM over the course of 30 days. Surviving parasites showed a reduced sensitivity to the compound. The figure shows mean IC_{50} of Dd2-B2 (±SEM) parasites and three independent MMV693183-resistant Dd2-B2 bulk cultures (Fl1, Fl2, Fl3) determined from two (FL1, FL2, FL3) to four (Dd2-B2) independent experiments measured in technical duplicates. Source data are provided as Source Data file.

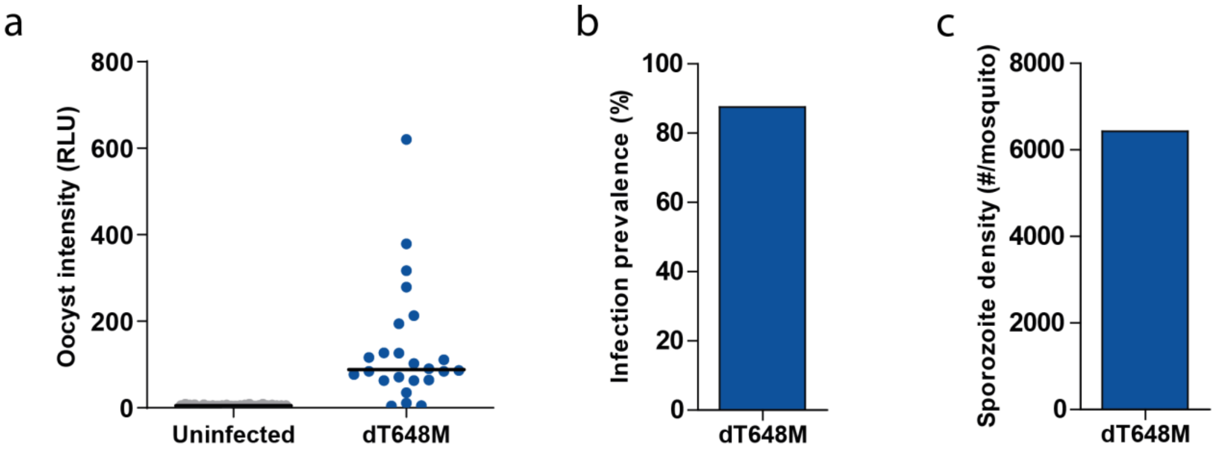

Supplementary Figure 8. Transmission of MMV693183-resistant *P. falciparum*. Mosquito infections of the drug-induced resistant mutant harboring a mutation in ACS (T648M). **a**, The oocyst intensity is depicted as relative luminescent units (RLU) (N = 24/group) (the horizontal bar indicates the median value), infection prevalence in 24 mosquitoes (**b**) and sporozoite density per mosquito of 42 dissected mosquitoes (**c**) after mosquito feeds. Source data are provided as Source Data file.

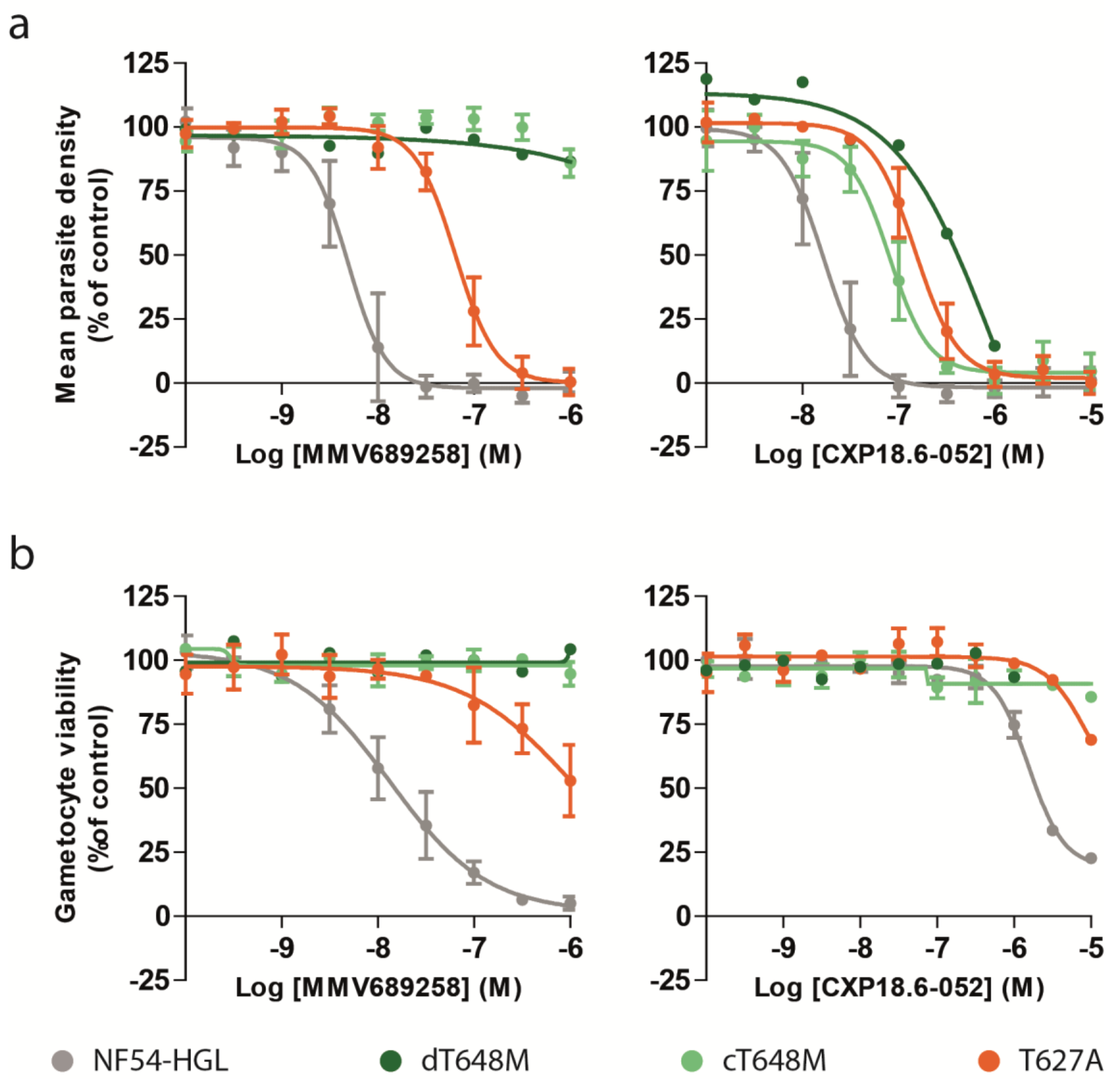

Supplementary Figure 9. Cross-resistance of AcAS mutants to pantothenamides. **a-b**, Drug sensitivity assays are performed on asexual (**a**) or sexual blood-stage parasites (**b**) with a T648M or T627A mutation in AcAS. The MMV693183-induced resistant parasite line (dT648M) was exposed to MMV689258 and CXP18.6-052 (1) in one experiment with two technical replicates and the CRISPR-engineered parasites (cT648M and T627A) were exposed to MMV689258 or CXP18.6-052 in three independent experiments (two technical replicates per experiment). The average value to control ± SEM is presented. Source data are provided as Source Data file.

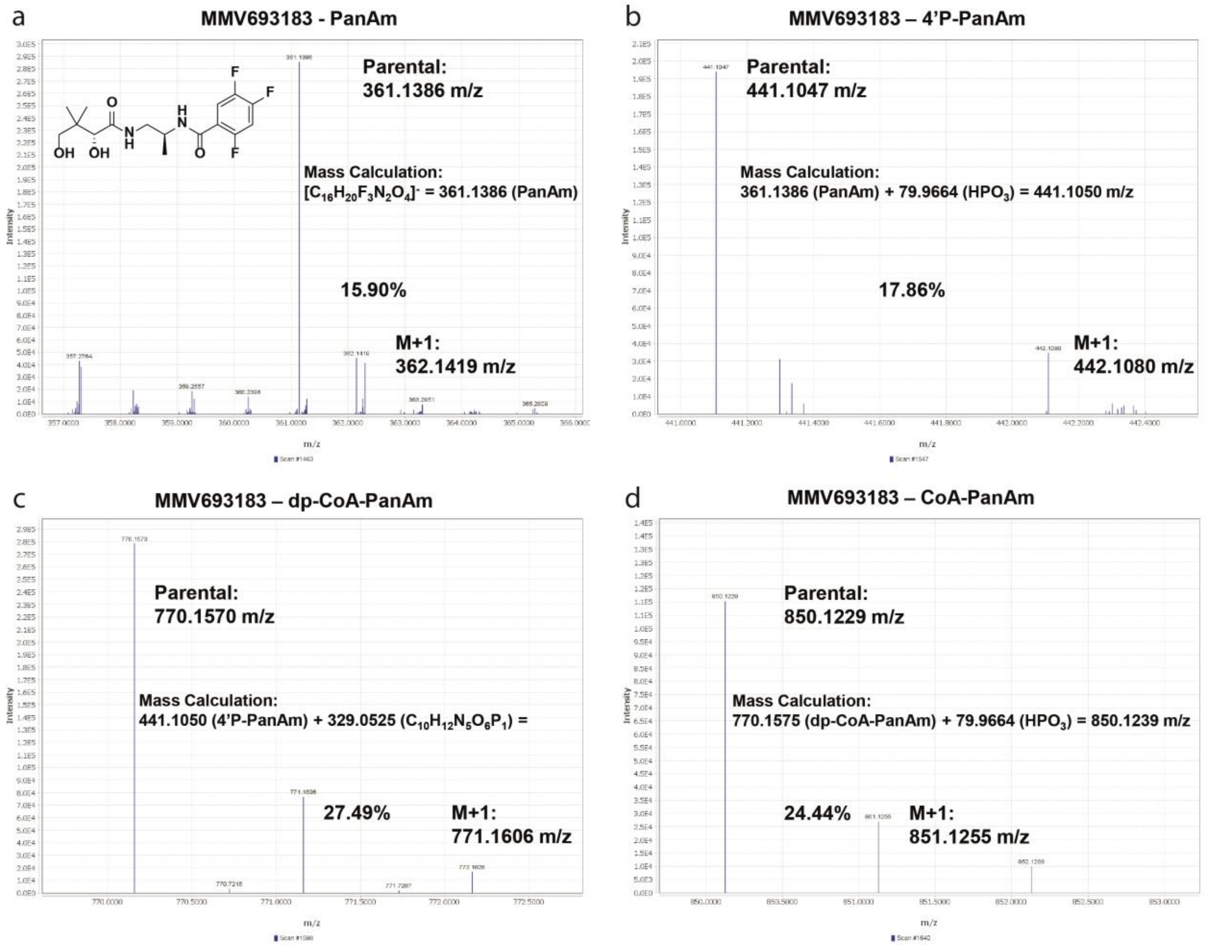

Supplementary Figure 10. Representative mass spectra of MMV693183 CoA-analogs. Mass spectra depicting the parental mass peak, the next heaviest isotope, and their relative abundance are depicted for the parent PanAm MMV693183 (**a**), the 4'-phospho-PanAm (4'P-PanAm), (**b**), the dephospho-CoA-PanAm (dp-CoA-PanAm), (**c**), and the CoA-PanAm, (**d**). Spectra were obtained in negative mode from a sample of human blood treated with 240 nM MMV693183 for 2.5 hours. The theoretical mass is presented by adding the expected monoisotopic mass of the individual elements to the mass of the preceding antimetabolite.

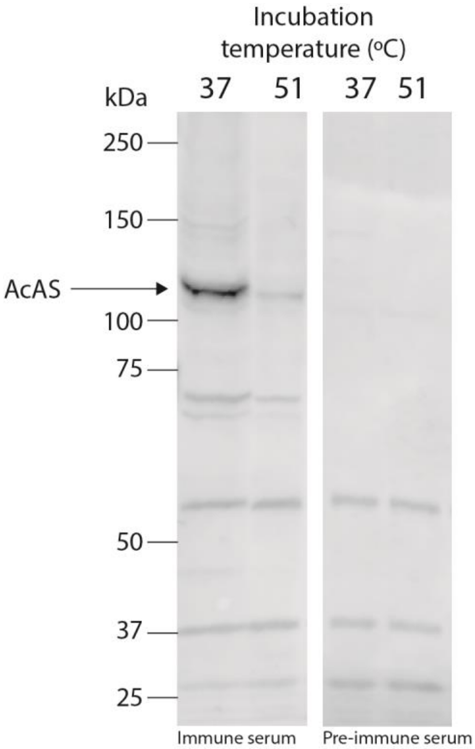

Supplementary Figure 11. Reactivity of immune serum used to extract ACS from parasite lysate. Western blot of NF54-HGL parasites incubated with rabbit pre-immune or immune sera directed against a recombinant AcAS fragment. Parasites were MACS-purified and incubated for 3 min at 37°C or 51°C, followed by incubation at 4°C for both conditions. Subsequently, parasites were lysed using lysis buffer for CETSA assays, three freeze-thaw cycles, and trituration through a 25G needle (N = 1). About 4×10^6 cells were loaded per lane and the blot was either incubated with immune serum (left) or pre-immune serum (right). The intensity of the non-specific bands is an indicator of equal loading. AcAS is indicated by the arrow and is predicted to be ~113.8 kDa.

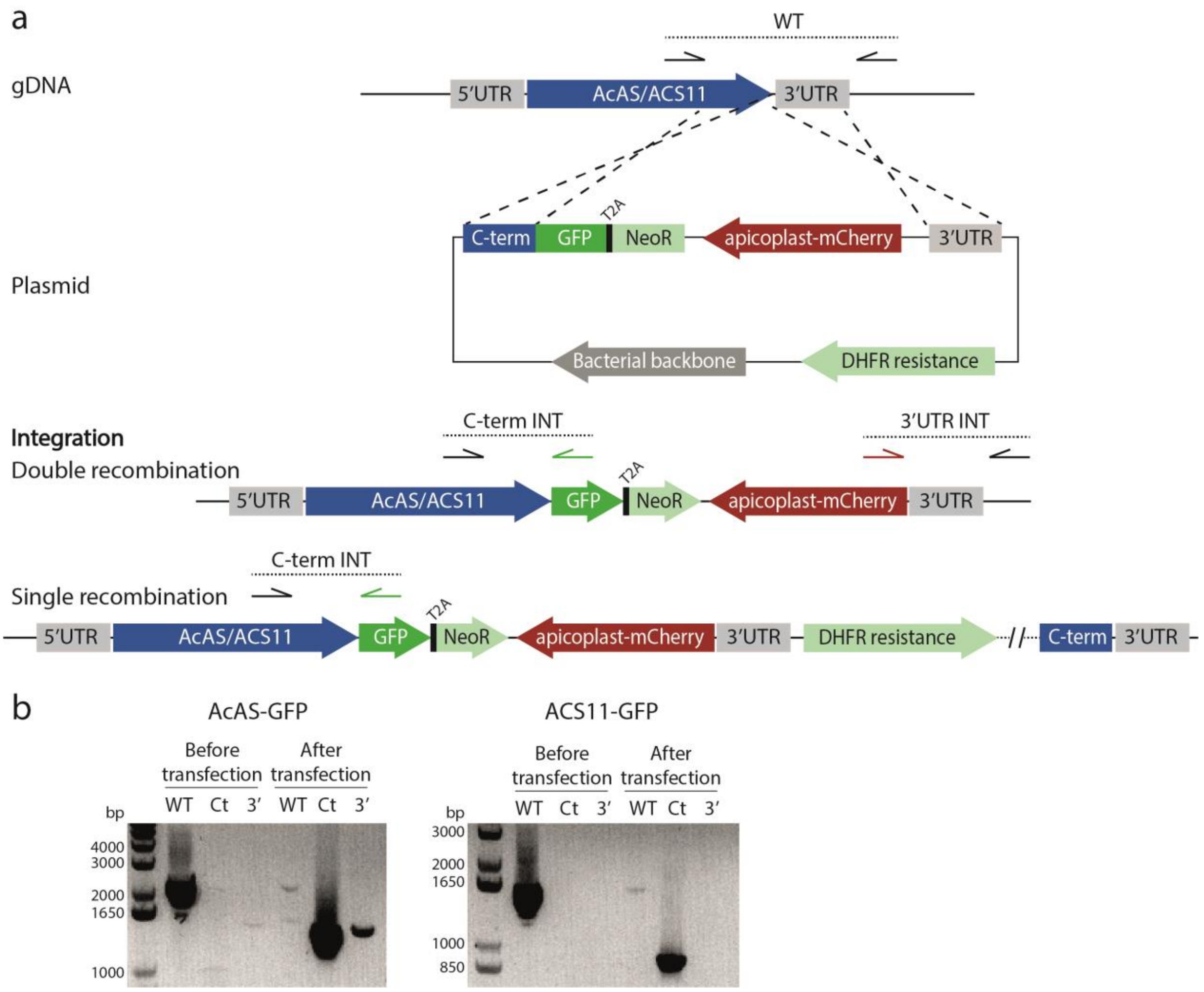

Supplementary Figure 12. Generation of AcAS and ACS11 fusion with GFP. a, Schematic of the GFP-tagging approach of *AcAS* and *ACS11* using selection linked integration. There are two possibilities for integration, double and single recombination, and PCR products are indicated for wild-type or integration. **b,** Diagnostic PCR confirmed that *AcAS* and *ACS11* are both fused to GFP. The plasmid is integrated in *ACS11* only by single recombination, while it is integrated by double recombination, and possibly single recombination, in *AcAS* (N = 1).

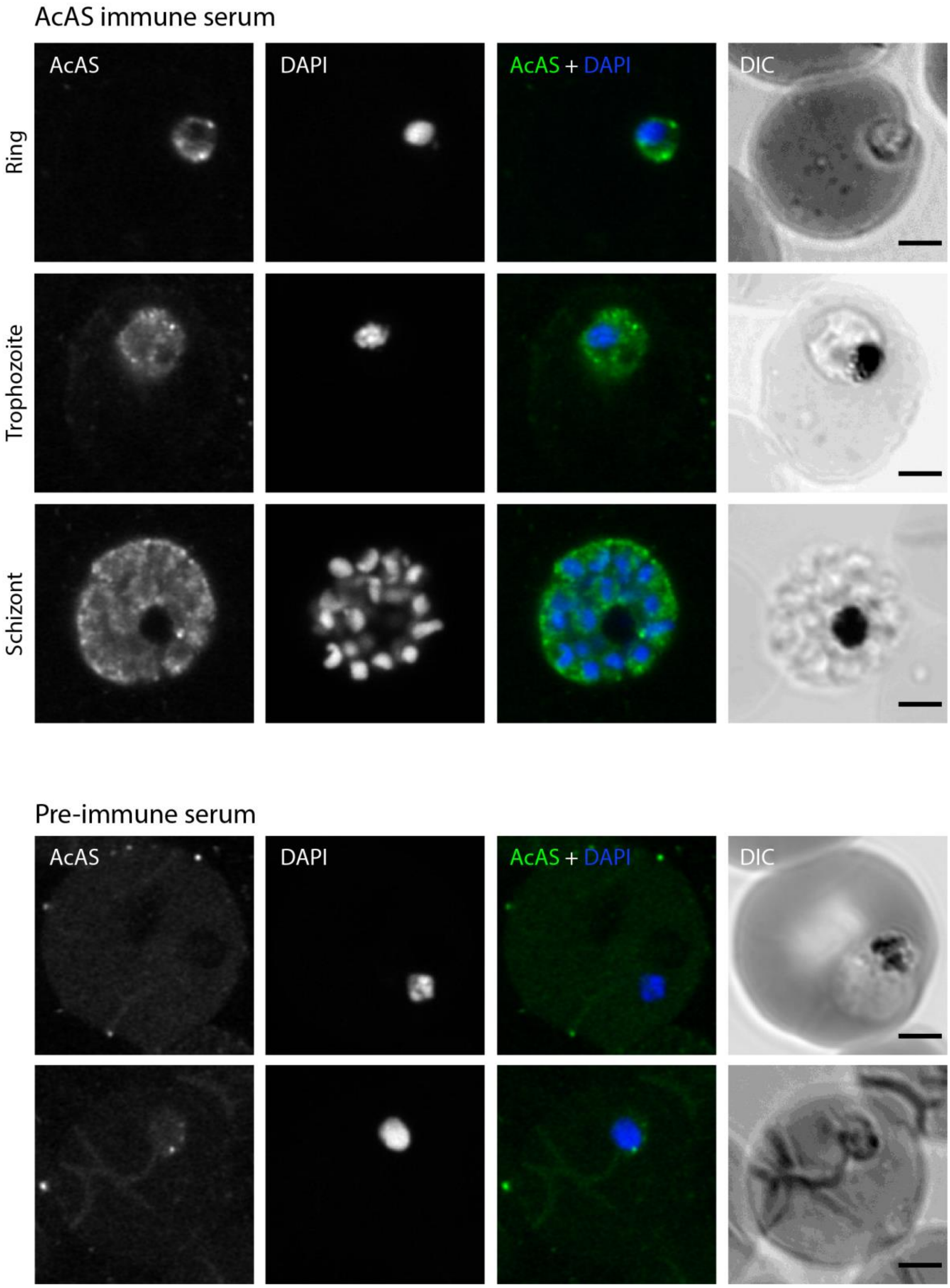

Supplementary Figure 13. Immunofluorescence microscopy of wild-type *Plasmodium falciparum* strain NF54 asexual blood-stage parasites stained with AcAS pre-immune or immune serum. Depicted are representative images of parasites stained with rabbit AcAS immune serum (top) or pre-immune serum (bottom). DNA was stained with DAPI. Scale bars, 2 µm.

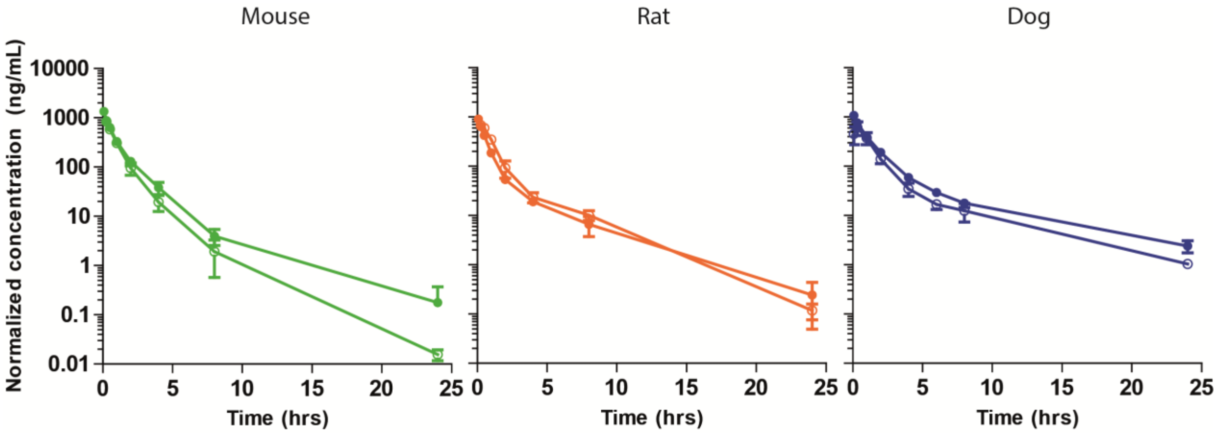

Supplementary Figure 14. Pharmacokinetics in mice, rats and dogs. The plasma concentration (±SD) of MMV693183 in mice, rats and dogs was determined and is presented as the mean concentration of compound normalized to the dose received (N = 3/treatment). Filled circles represent intravenous injection (3 mg/kg for mice and rats, 1 mg/kg for dogs) and clear circles represent *per os* dosing (30 mg/kg for mice and rats, 2 mg/kg for dogs). Source data are provided as Source Data file.

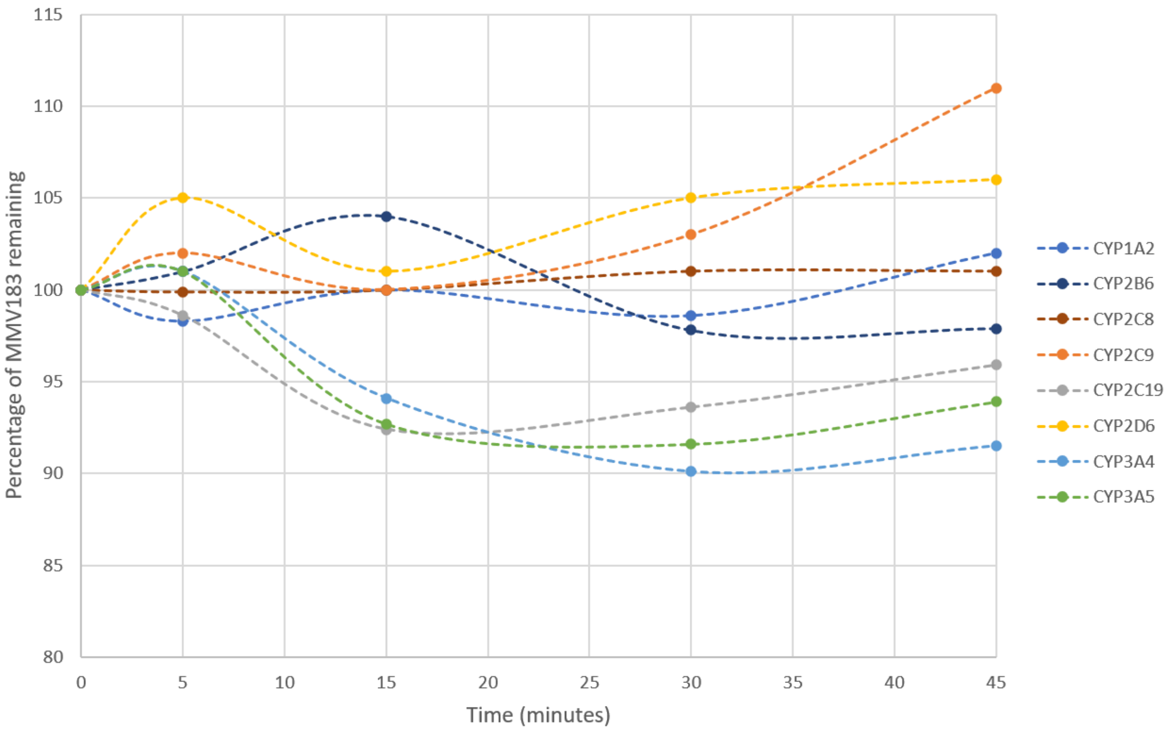

Supplementary Figure 15. Percentage of MMV693183 remaining after incubation with recombinant human CYPs. Human recombinant CYP enzymes were incubated with MMV693183, and the remaining concentration was quantified over time. Source data are provided as Source Data file.

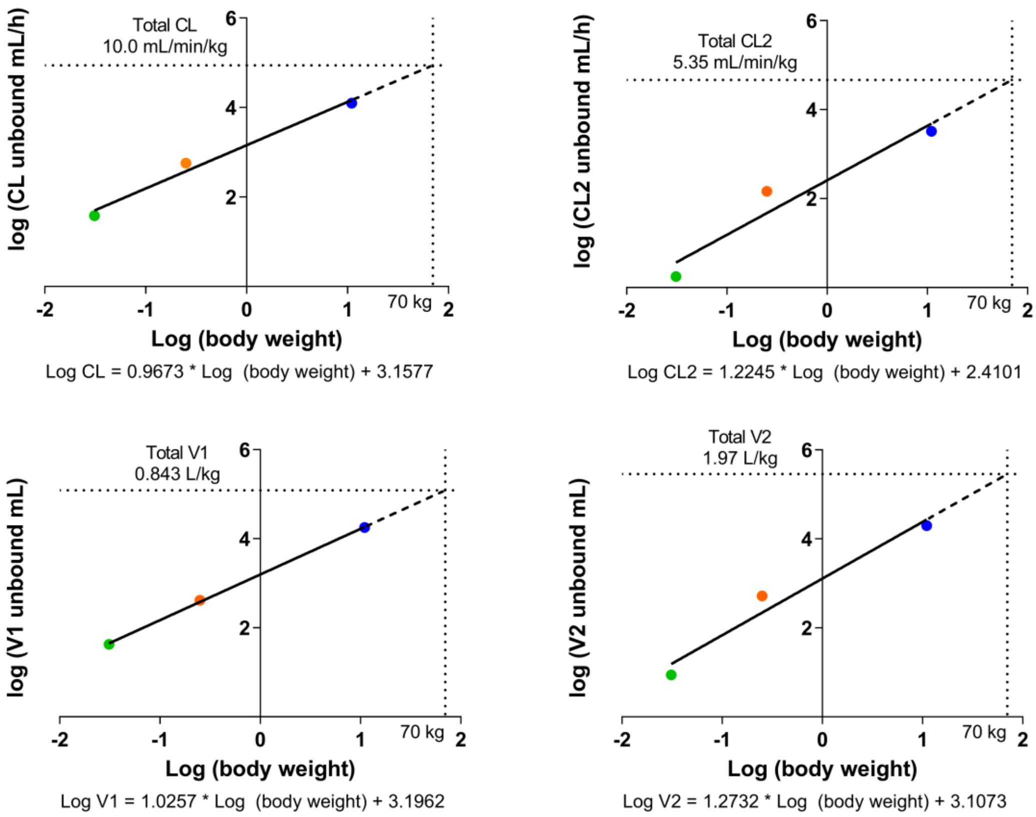

Supplementary Figure 16. Prediction of human pharmacokinetic values using allometry scaling. The average unbound plasma concentration was calculated in Phoenix 64 using a two-compartment model with multiplicative weighting parameterized by clearance and volume of distribution in the central (CL; V1) or effect (CL2; V2) compartment in mice (green), rats (orange) and dogs (blue). Allometry scaling is used to predict the human parameters.

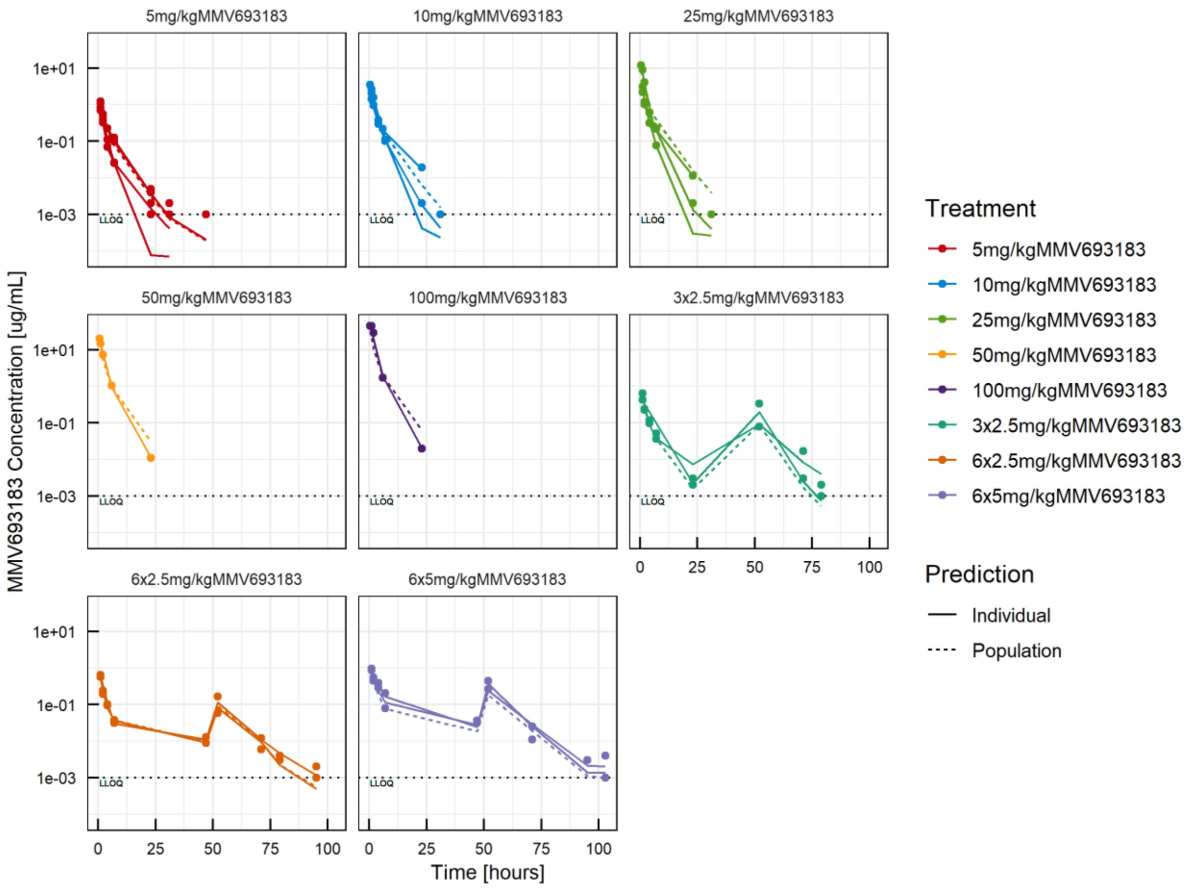

Supplementary Figure 17. PK individual fits from humanized mice. PK data in NSG mice were calculated using a non-linear mixed effects (NLME) approach. A 3-compartment model with zero-order absorption, linear elimination and a combined residual unexplained variability was selected as the best PK model describing the PK data. There was no lag time observed for drug absorption. Observations are represented by full circles, continuous lines represent individual predictions, while dashed lines represent the PK model at a population level. The lower limit of quantification (LLOQ) was 0.001 µg/ml. Observed concentrations below the LLOQ were represented at the LLOQ value to allow graphical representation. 3x2.5 mg/kg: 3-day regimen dose of 2.5 mg/kg once per day; 6x2.5 mg/kg: 3-day regimen of 2.5 mg/kg twice per day; 6x5 mg/kg: 3-day regimen of 5 mg/kg twice per day. LLOQ: lower limit of quantification. Source data are provided as Source Data file.

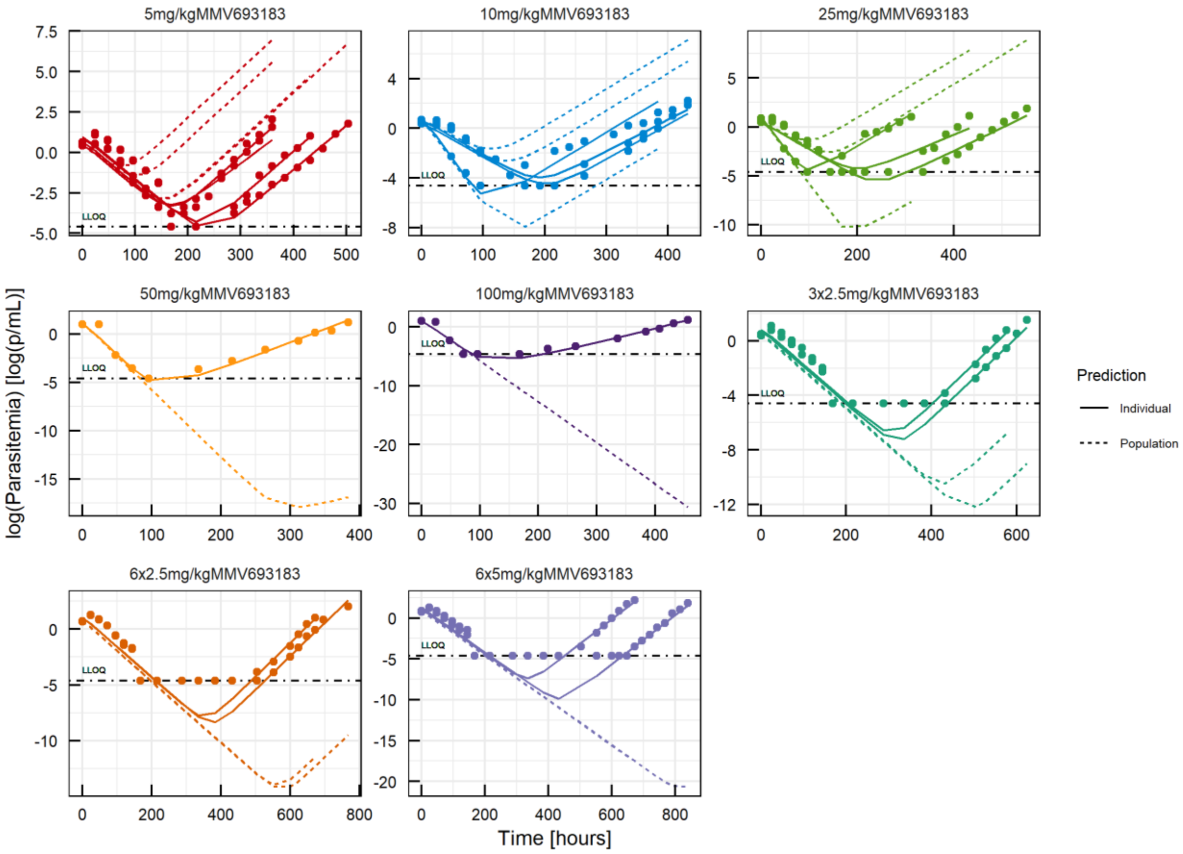

Supplementary Figure 18. PD individual fits using the *in vitro* clearance model. Observations are represented by full circles, continuous lines represent individual predictions, while dashed lines represent the PK model at a population level. Source data are provided as Source Data file.

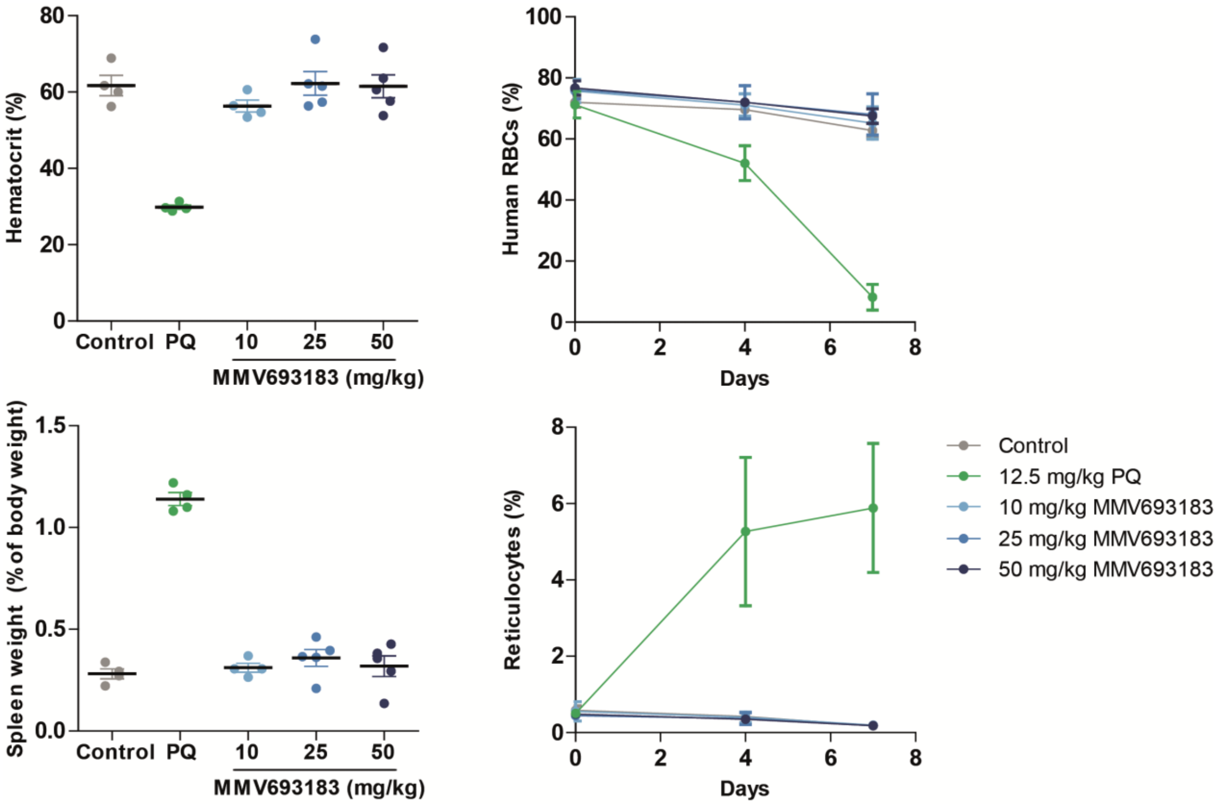

Supplementary Figure 19. Absence of detectable hemolytic toxicity in G6PD-deficiency mouse model. The hemolytic activity of MMV693183 was determined in NSG mice engrafted with human RBCs from an A- G6PD-deficient donor with primaquine as a positive control. The mean percentage hematocrit, human RBCs and reticulocytes, and the spleen weight normalized to body weight ±SD are shown for N = 4 mice/treatment (Control, PQ, 10 mg/kg MMV693183) or 5 mice/treatment (25 mg/kg, 50 mg/kg MMV693183). Source data are provided as Source Data file.

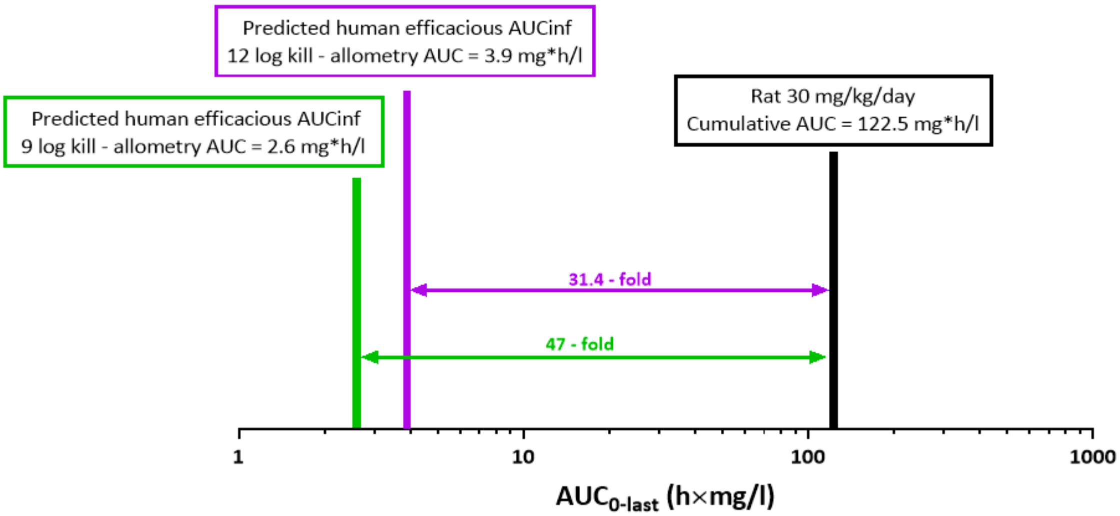

Supplementary Figure 20. Safety margins. The figure shows cumulative exposure in rats treated at 30 mg/kg/day for eight days versus a conservative estimate of human efficacious exposure, using allometry-based predictions of human clearance, to achieve either a 9 or 12 log reduction in parasitemia.

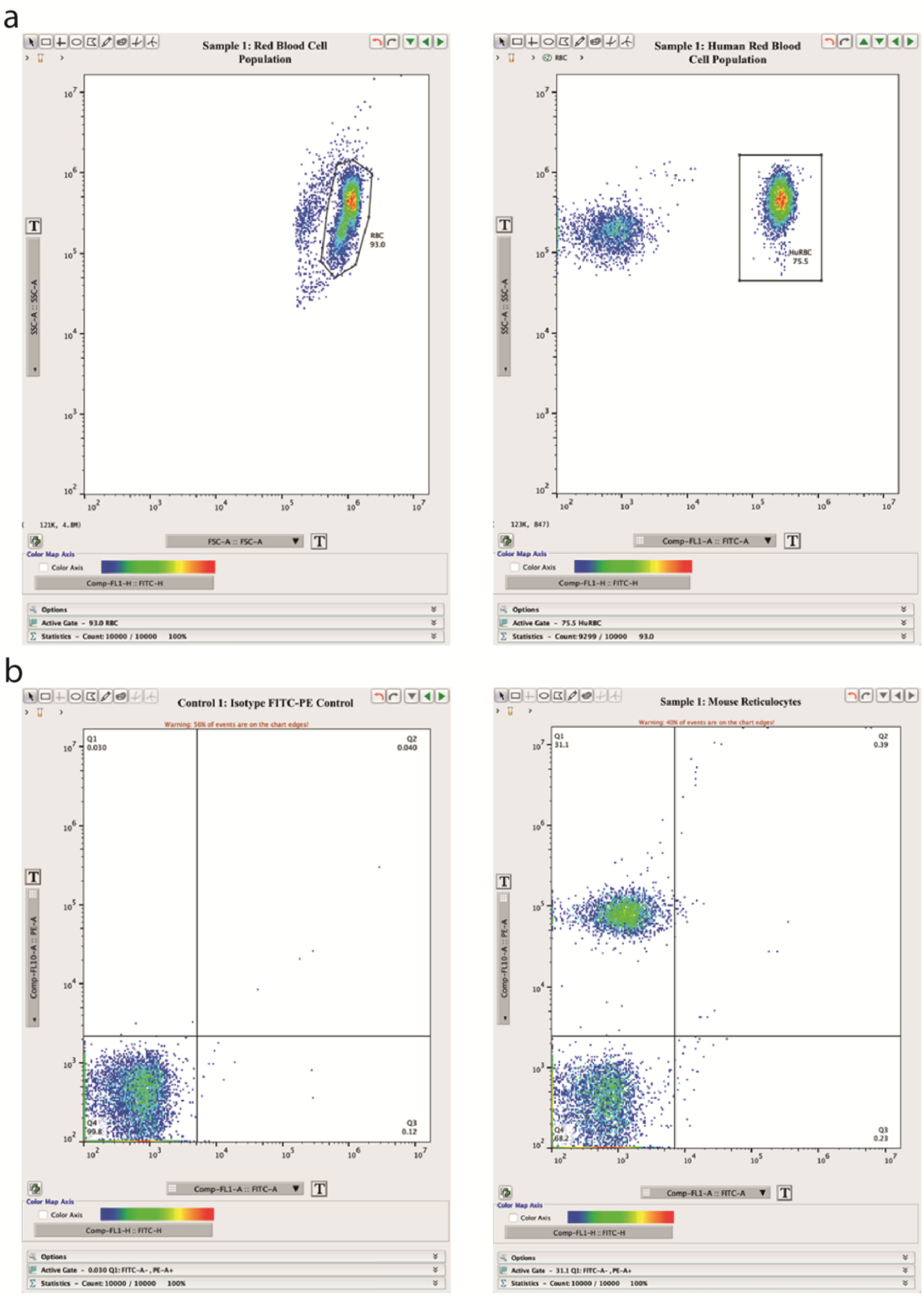

Supplementary Figure 21. Gating strategy to determine hemolytic toxicity. a, gating strategy for human RBCs quantification. Blood samples were stained with anti-glycophorin A (FITC). RBCs are gated based on the FSC-A and SSC-A (left), subsequently human RBCs (HuRBCs) are selected based on their positive signal in the FITC channel in the RBC population (right). **b**, gating strategy to quantify mouse reticulocytes. Blood samples were stained with anti-mouse TER119 (PE) and anti-mouse CD71 (FITC). From a RBC population (a, left), quadrants were made in the control samples stained with isotype controls (left). Mouse reticulocytes are presented in Q3 (right).

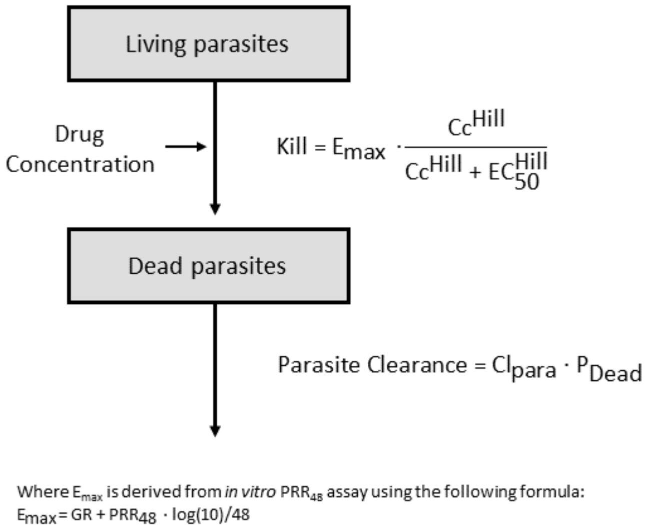

Supplementary Figure 22. Description of the *in vitro* clearance model used to fit the NSG mice data.

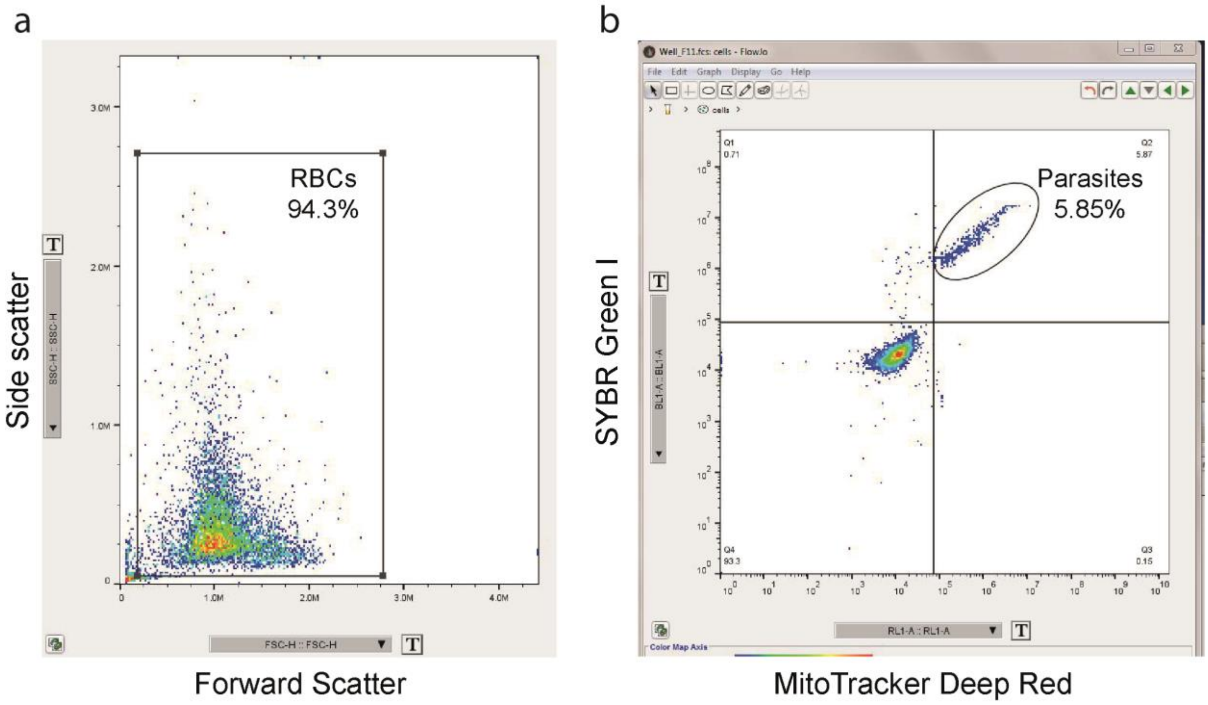

Supplementary Figure 23. Gating strategy for flow cytometry-based analysis of *Plasmodium falciparum* parasitemias. Intra-erythrocytic parasites were cultured at a starting parasitemia of 0.3% and 1% hematocrit for 72 h in the presence of a range of drug concentrations, with no-drug controls. Cells were then labeled with 1X SYBR Green I and 100 nM MitoTracker Deep Red (ThermoFisher) and parasitemias measured by flow cytometry using a BD Accuri C6 PLus flow cytometer. a, Forward and side scatter gating of the total red blood cell (RBC) population. b, SYBR Green I and MitoTracker Deep Red gating of the RBC population, showing parasitized RBCs in the top right gate (5.85% parasitemia). Data show a control gating of untreated parasite cultures from a representative assay testing MMV693183 dose-response profiles of the humanized mouse-adapted *P. falciparum* 3D7 line.

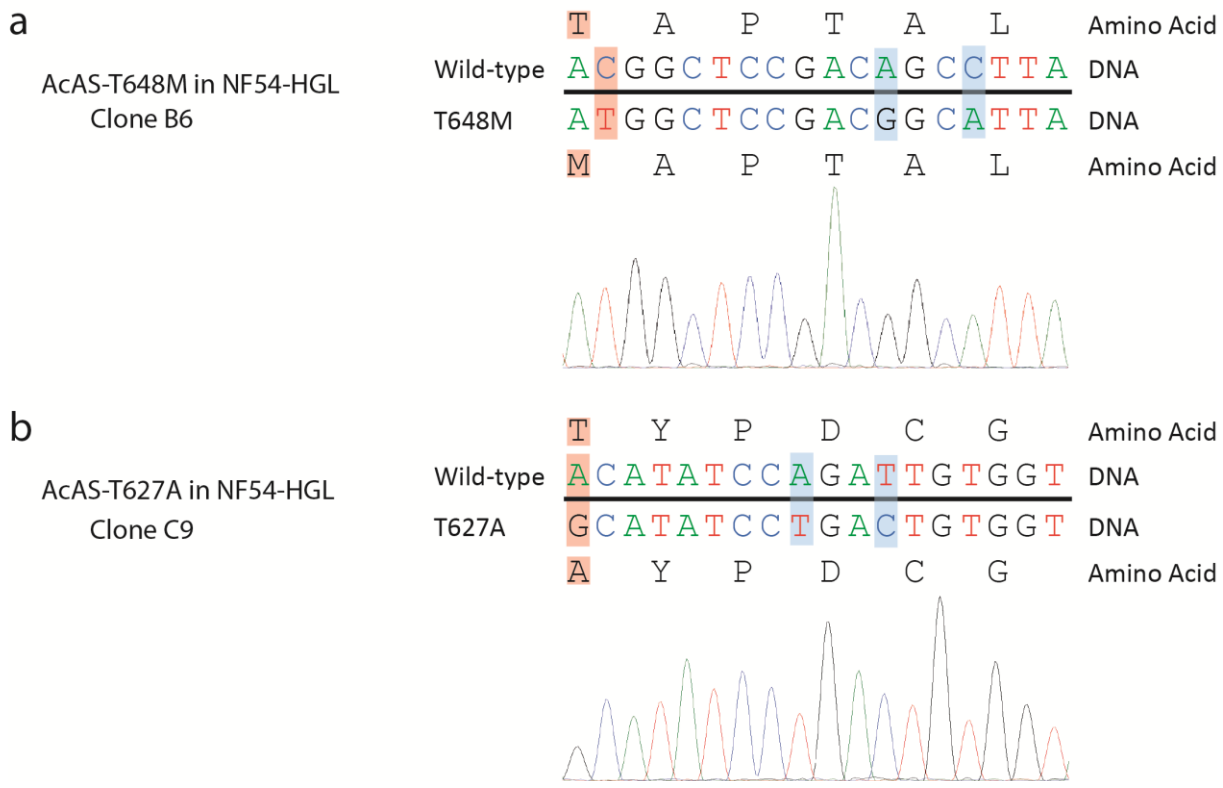

Supplementary Figure 24. Sequence verification of CRISPR-Cas9 engineered mutants. The *AcAS*-T648M (**a**) and the *AcAS*-T627A (**b**) mutation were introduced in NF54-HGL parasites and the integration was confirmed by Sanger sequencing.

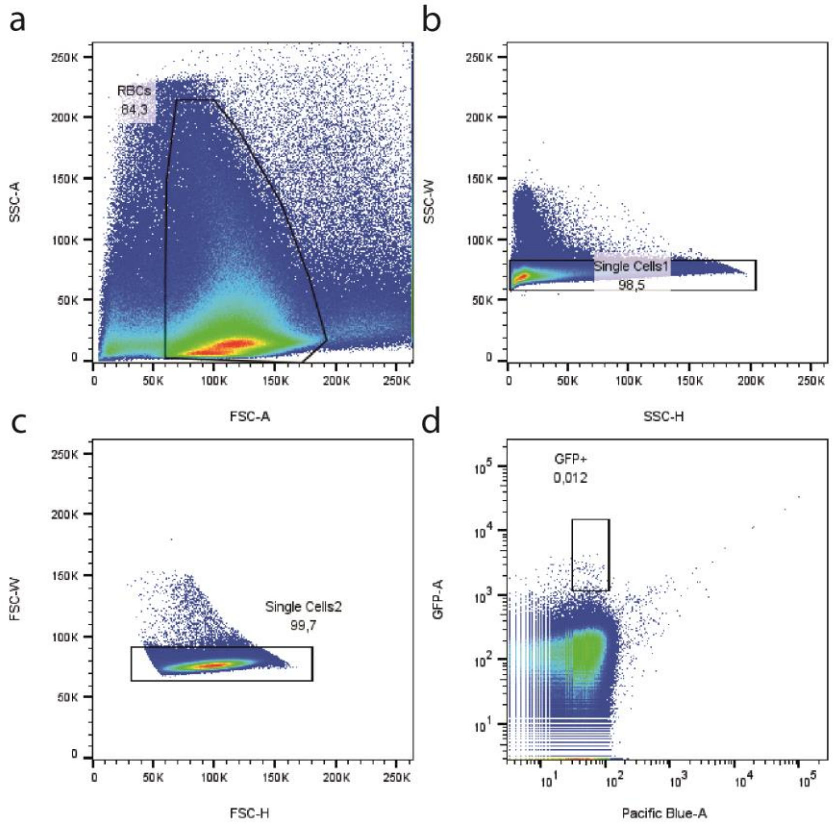

Supplementary Figure 25. Gating strategy to sort endogenously tagged parasites. a, An RBC population was selected based on the FSC-A and SSC-A plot. **b,** Subsequently, single cells 1 were selected from the SSC-H, SSC-W plot, **c,** followed by single cells 2 from the FSC-H, FSC-W plot. **d,** From this final population, GFP-positive cells were sorted.

Supplementary Table 1. Modification of the left hand side, linker and amide

Structural modification/variation	Structure	Compound code	IC_{50} (µM)
Early candidate	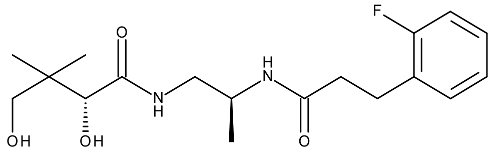	MMV689258	0.007
bioisosteres	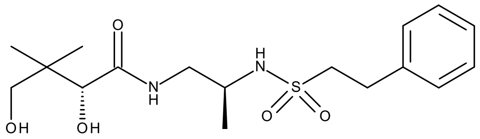	MMV692748	>10
bioisosteres	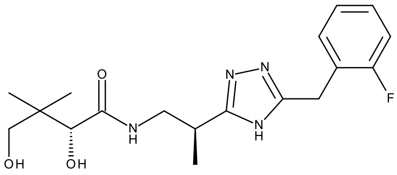	MMV693175	>10
bioisosteres	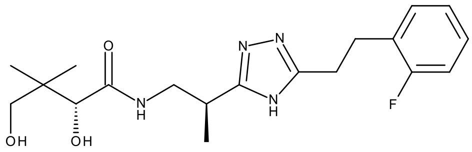	MMV884788	0.554
linker length between amides	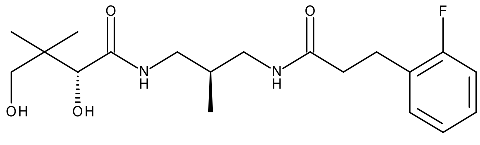	MMV6922003	>10
substitutions of the ethyl linker	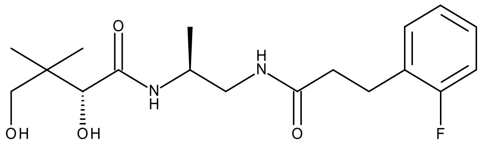	MMV689041	7.9
substitutions of the ethyl linker	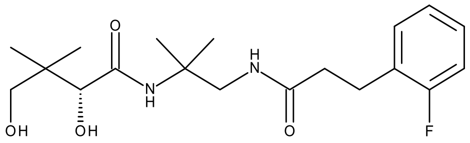	MMV689261	>10
substitutions of the ethyl linker	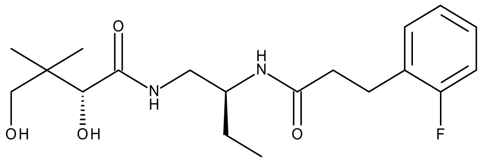	MMV692676	0.122
substitutions of the ethyl linker	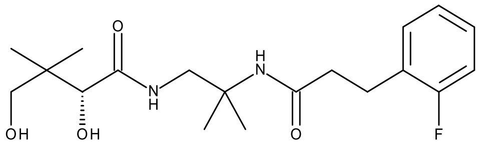	MMV689455	1.19
primary hydroxyl	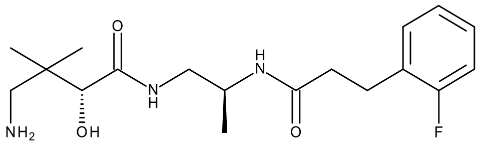	MMV1545511	>1

primary hydroxyl	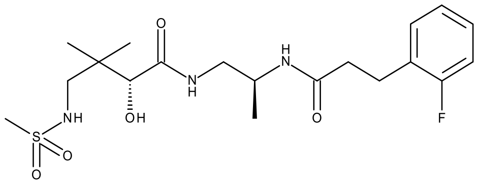	MMV1545779	>1
geminal dimethyl	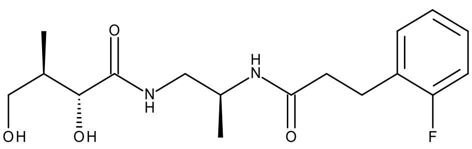	MMV884790	>1
geminal dimethyl	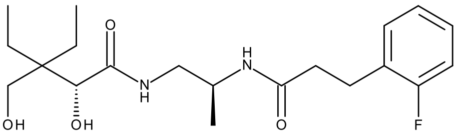	MMV884964	>1
secondary hydroxyl	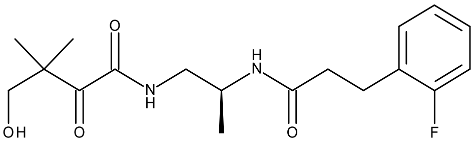	MMV1545786	1
secondary hydroxyl	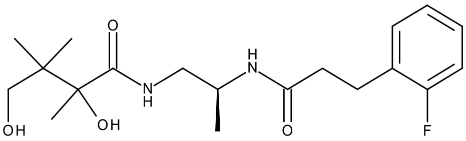	MMV1545785	1
stereochemistry	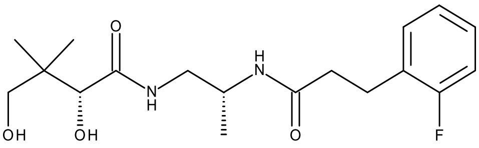 R,R enantiomer	MMV689260	0.21
stereochemistry	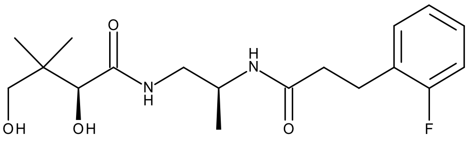 S,S enantiomer	MMV884787	0.235
stereochemistry	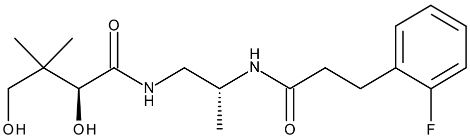 S,R enantiomer	MMV884968	>1

IC_{50} was determined in asexual blood stages NF54 parasites.

Supplementary Table 2. Modifications of the right hand side

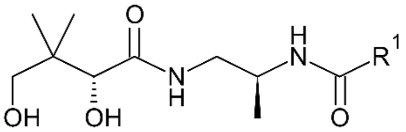

Compound code	Modification	Modification at position					IC_{50} (µM)
	Aryl 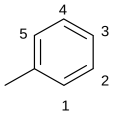	1	2	3	4	5	
MMV154200	Aryl	F					0.041
MMV892807	Aryl	Cl					> 10
MMV892812	Aryl	OMe					> 10
MMV892613	Aryl	CF3					> 10
MMV1542005	Aryl	Me					> 10
MMV1542001	Aryl		F				0.0024
MMV892808	Aryl		Cl				0.0023
MMV892811	Aryl		OMe				0.032
MMV892614	Aryl		CF3				0.143
MMV1542099	Aryl		Me				0.022
MMV884962	Aryl		CN				0.0074
MMV884970	Aryl		NMe2				> 10
MMV884969	Aryl		SO2Me				> 10
MMV1542002	Aryl			F			0.082
MMV892809	Aryl			Cl			0.103
MMV892810	Aryl			OMe			0.344
MMV892612	Aryl			CF3			0.132
MMV1542100	Aryl			Me			0.085
MMV884961	Aryl			CN			0.535
MMV1576723	Aryl	F	F				0.0327
MMV1576724	Aryl	F			F		0.007
MMV693185	Aryl	F				F	0.478
MMV977411	Aryl	F	Cl				0.012
MMV977410	Aryl	F	CN				0.019
MMV1576728	Aryl	F			Cl		0.0026
MMV693182	Aryl	F			CN		0.0075
MMV1576731	Aryl		F	F			0.0046
MMV977407	Aryl		Cl	F			0.0034
MMV1576725	Aryl		F	Cl			0.056
MMV977409	Aryl		CN	F			0.084
MMV693184	Aryl	F	F	F			0.037
MMV693183	Aryl	F		F	F		0.0028
MMV692009	Aryl	F		F	Cl		0.0023
MMV893655	Aryl	F		Cl	Cl		0.0197
MMV893115	Aryl	F		F	Me		0.066
MMV893337	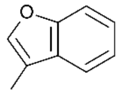						0.0039

MMV893335	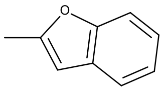						0.0125
MMV893340	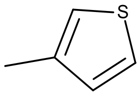						0.0146
MMV893344	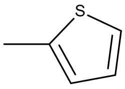						0.0182
MMV893336	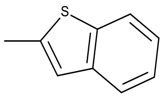						0.0199
MMV893375	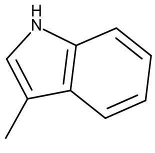						0.041
MMV893339	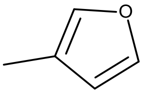						0.042
MMV893121	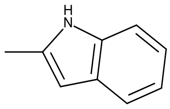						0.053
MMV893338	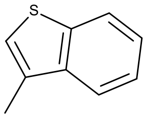						0.195
MMV893376	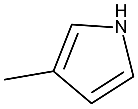						1.8
MMV893345	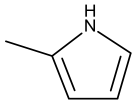						> 10
MMV892814	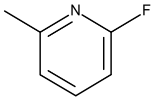						> 10

IC_{50} was determined in asexual blood stages NF54 parasites.

Supplementary Table 3. PK parameters derived from the data shown in Fig 1

Compound	T_{max} (h)	$T_{1/2}$ (h)	C_{max} (ng/ml)	$AUC_{0\text{-}24h}$ (h*ng/ml)
MMV689258	1.00	3.26	3780	6730
MMV693183	1.50	2.49	12900	33700
MMV884962	1.00	3.32	3920	8650
MMV693182	1.00	3.19	5270	15100
MMV976394	1.00	2.95	5810	10800
MMV1542001	1.00	2.80	5150	11800

Supplementary Table 4. Solubility of MMV693183

Solution	Solubility (µM)
**PBS**	19.7
**FaSSIF**	25.5
**FeSSIF**	25.0

Source data are provided as Source Data file.

Supplementary Table 5. Selection of MMV693183-resistant parasites in *P. falciparum*.

Inoculum and strain	Concentration MMV693183 for selection	Frequency (# of parasite positive flasks or wells)	IC_{50} shift	Mutation
$1x10^5$ Dd2-B2	15 nM	0/24 wells	N/A	N/A
$1x10^7$ Dd2-B2	15-22 nM (1x)<br>10.5-14 nM (2x)	0/3 flasks	N/A	N/A
$1x10^8$ Dd2-B2	10.5-14 nM	0/3 flasks	N/A	N/A
$1x10^9$ Dd2-B2	12-27 nM	3/3 flasks	13x	Single SNP T648M in PF3D7_0627800 (AcAS) (3/3)
NF54	7 nM	2/2 flasks	77x	T648M in PF3D7_0627800 (AcAS) (2/2)

Supplementary Table 6. Mutations in MMV693183-resistant NF54 parasites

Chromosome	Position	GeneID	Annotation	Mutation
PF3D7_04_v3	374669	PF3D7_0407600	Conserved *Plasmodium* protein, unknown function	Non_Synonymous_Coding (ATA->ACA I541T; I544T)
PF3D7_05_v3	1332563			NonCoding - upstream
PF3D7_06_v3	52711	PF3D7_0601400	Erythrocyte membrane protein 1 (PfEMP1), pseudogene	Stop_Gained (TTG->TAG L454*)
PF3D7_06_v3	1115595	PF3D7_0627800	Acetyl-CoA synthetase	Non-Synonymous_Coding (ACT->ATG T648M)
PF3D7_09_v3	805836	PF3D7_0919800	TLD domain-containing protein	Codon_Change_Plus_Codon_Insertion TTATTA insert disrupting AA 606
PF3D7_14_v3	207110	PF3D7_1405900	RNA-binding protein, putative	Non_Synonymous_coding (GAT->CAT D518H)
PF3D7_14_v3	2029231			Noncoding - upstream

Supplementary Table 7. Pharmacokinetic parameters in mice derived from data shown in Supplementary Figure 14

Dose	Route	Parameters	Units	Animal1	Animal2	Animal3	Mean	SD
3	i.v.	K_{el}	1/hr	0.59	0.55	0.61	**0.58**	0.03
3	i.v.	$T_{1/2}$	hr	1.17	1.26	1.14	**1.19**	0.06
3	i.v.	C_0	ng/ml	4944.5	5144.1	4952.3	**5013.7**	113.0
3	i.v.	AUC_{last}	hr*ng/ml	3750.0	4121.9	3419.4	**3763.8**	351.5
3	i.v.	$AUC_{0-\infty}$	hr*ng/ml	3752.1	4122.3	3419.8	**3764.7**	351.4
3	i.v.	CL_{total}	ml/min/kg	13.33	12.13	14.62	**13.36**	1.25
3	i.v.	Vss_obs	ml/kg	1079.1	1028.1	972.3	**1026.5**	53.4
30	p.o.	K_{el}	1/hr	0.58	0.68	0.74	**0.67**	0.08
30	p.o.	$T_{1/2}$	hr	1.20	1.02	0.93	**1.05**	0.14
30	p.o.	T_{max}	hr	0.25	0.25	0.25	**0.25**	0.00
30	p.o.	C_{max}	ng/ml	26100.0	25500.0	23700.0	**25100.0**	1249.0
30	p.o.	AUC_{last}	hr*ng/ml	29452.7	27128.8	22505.2	**26362.2**	3536.6
30	p.o.	$AUC_{0-\infty}$	hr*ng/ml	29453.4	27129.7	22505.7	**26362.9**	3536.7
30	p.o.	CL_{total}	ml/min/kg	16.98	18.43	22.22	**19.21**	2.7
30	p.o.	%F	%	78.24	72.06	59.78	**70.03**	9.39

PK parameters were calculated using WinNonLin (Phoenix. Version 6.3). I.v.: intravenous; p.o.: per os.

Supplementary Table 8. Pharmacokinetic parameters in rats derived from data shown in Supplementary Figure 14

Dose	Route	Parameters	Units	Animal1	Animal2	Animal3	Mean	SD
3	i.v.	K_{el}	1/hr	0.19	0.23	0.25	**0.22**	0.03
3	i.v.	$T_{1/2}$	hr	3.62	3.03	2.72	**3.13**	0.46
3	i.v.	C_0	ng/ml	3682.2	2966.7	3086.4	**3245.1**	383.2
3	i.v.	AUC_{last}	hr*ng/ml	2795.1	2252.0	2137.3	**2394.8**	351.4
3	i.v.	$AUC_{0-\infty}$	hr*ng/ml	2802.5	2254.2	2138.5	**2398.4**	354.7
3	i.v.	CL_{total}	ml/min/kg	17.84	22.18	23.38	**21.14**	2.91
3	i.v.	Vss_obs	ml/kg	1930.8	1963.6	1992.7	**1962.4**	31.0
30	p.o.	K_{el}	1/hr	0.27	0.28	0.26	**0.27**	0.01
30	p.o.	$T_{1/2}$	hr	2.56	2.49	2.65	**2.57**	0.08
30	p.o.	T_{max}	hr	0.25	0.25	0.25	**0.25**	0.00
30	p.o.	C_{max}	ng/ml	21800.0	17200.0	22400.0	**20466.7**	2844.9
30	p.o.	AUC_{last}	hr*ng/ml	33270.0	23500.9	30420.9	**29063.9**	5023.9
30	p.o.	$AUC_{0-\infty}$	hr*ng/ml	33282.9	23509.2	30439.3	**29077.1**	5027.3
30	p.o.	CL_{total}	ml/min/kg	15.02	21.27	16.43	**17.57**	3.28
30	p.o.	%F	%	138.77	98.02	126.92	**121.24**	20.96

PK parameters were calculated using WinNonLin (Phoenix. Version 6.3). I.v.: intravenous; p.o.: per os.

Supplementary Table 9. Pharmacokinetic parameters in dogs derived from data shown in Supplementary Figure 14

Dose	Route	Parameters	Units	Animal1	Animal2	Animal3	Mean	SD
1	i.v.	$T_{1/2}$	hr	2.15	5.69	4.83	4.23	1.85
1	i.v.	C_{max}	ng/ml	1180	892	1160	1077	161
1	i.v.	AUC_{last}	hr*ng/ml	1302	1514	1452	1423	109
1	i.v.	$AUC_{0-\infty}$	hr*ng/ml	1347	1540	1466	1451	97.4
1	i.v.	CL_{total}	ml/min/kg	12.37	10.82	11.37	11.52	0.787
1	i.v.	Vss_obs	L/kg	1.33	2.19	1.85	1.79	0.435
2	p.o.	$T_{1/2}$	hr	2.11	4.97	1.70	2.93	1.78
2	p.o.	T_{max}	hr	0.50	0.25	0.25	0.333	0.25-0.50
2	p.o.	C_{max}	ng/ml	1630	1230	997	1286	320
2	p.o.	AUC_{last}	hr*ng/ml	1942	1864	1727	1844	109
2	p.o.	$AUC_{0-\infty}$	hr*ng/ml	2055	1880	1772	1902	143
2	p.o.	F	%	68.5	59.9	62.9	63.7	4.99

i.v.: intravenous; p.o.: per os.

Supplementary Table 10. Caco-2 permeability assay

Compound	Average Papp x10^{-6}/sec		
	**A to B**	**B to A**	**Efflux ratio**
MMV693183	2.9	13.5	4.7
Furosemide	0.2	12.9	67.1
Atenolol	0.4	0.4	1.0
Verapamil	19.0	40.3	2.1
Carbamazepine	25.0	28.9	1.2
Domperidone	3.5	25.2	7.3

Apical/Basal pH: 7.4/7.4. Source data are provided as Source Data file.

Supplementary Table 11. ***In vitro*** **ADME parameters of MMV693183**

	**Mouse**	**Rat**	**Dog**	**Human**
Fu,p	0.666	0.601	0.587	0.481
B:P	0.98	0.91	N.D.	0.97
In vitro hepatocyte CL_{int} (µl/min/10^6 cells) (SE)	1.12 (2.02)	3.35 (0.610)	4.99 (1.17)	0.40*

*Data derived from Table 1. Source data are provided as Source Data file.

Supplementary Table 12. Metabolite identification in an *in vitro* human hepatocyte relay assay

**Name**	**RT (min)**	**Ion (m/z)**	**Metabolism**	***In vitro* (MS response)**
Parent	5.26	363	-	85%
M1	5.20	377	Oxidation	9%
M2	4.90	539	Glucuronide	2%
M3	4.69	383 (Na adduct)	Dehydrogenation	2%
M4	5.03	383 (Na adduct)	Dehydrogenation	2%
M5	4.69	539	Glucuronide	<1%
M6	4.04	343	Possibly loss of H_2O	<1%
M7	4.21	443	Sulphate	<1%
M8	4.59	539	Glucuronide	<1%
M9	3.4.79	539	Glucuronide	<1%

RT: retention time

Supplementary Table 13. Metabolite identification in dog plasma and urine samples

Name	Ion (m/z)	RT (min)	Metabolism	Formula	Plasma	Urine
Parent	362	3.36	-	$C_{16}H_{21}F_3N_2O_4$	Yes	Yes
M1	M+174	2.89	Glucuronide + dehydrogenation	$C_{22}H_{27}F_3N_2O_{10}$	No	Yes
M2	M+16	2.96	Oxidation	$C_{16}H_{21}F_3N_2O_5$	No	Yes
M3	M+176	3.02	Glucuronide	$C_{22}H_{29}F_3N_2O_{10}$	No	Yes
M4	M+174	3.07	Glucuronide and Dehydrogenation	$C_{22}H_{27}F_3N_2O_{10}$	No	Yes
M5	M+176	3.14	Glucuronide	$C_{22}H_{29}F_3N_2O_{10}$	Yes	Yes
M6	M-2	3.17	Dehydrogenation	$C_{16}H_{19}F_3N_2O_4$	Yes	Yes
M7	M+172	3.22	Glucuronide and didehydrogenation	$C_{22}H_{25}F_3N_2O_{10}$	No	Yes
M8	M-2	3.26	Dehydrogenation	$C_{16}H_{19}F_3N_2O_4$	Yes	Yes
M9	M+14	3.36	Oxidation and dehydrogenation	$C_{16}H_{19}F_3N_2O_5$	Yes	Yes
M10	M-4	3.65	Tetra-dehydrogenation	$C_{16}H_{17}F_3N_2O_4$	Yes	Yes

Supplementary Table 14. Renal excretion of MMV693183 in rats intravenously treated with 3 mg/kg

Parameters	Units	Animal1	Animal2	Animal3	Mean	SD
Stock concentration	mg/ml	1.5	1.5	1.5		
Animal weight	g	227	223	218		
Dose volume	µl	460	440	440		
Dose administered	µg	690	660	660		
Concentration in urine	ng/ml	27920	106400	15600		
Urine volume	ml	5.0	4.7	5.0		
Total urine excretion	µg	139.6	500.1	78		
Urine fraction		0.20	0.76	0.12	0.36	0.35

Source data are provided as Source Data file.

Supplementary Table 15. Renal excretion of MMV693183 in dogs intravenously treated with 1 mg/kg

	Intervals (h)	Concentration (ng/ml)	Collected volume (ml)	Total in urine (µg)	Dose in urine
**Animal 1**	0-4	2790	8	22.32	
	4-8	-	0	-	
	8-24	-	0	-	
	Total		8	22.32	0.002
**Animal 2**	0-4	4550	60	273.0	
	4-8	-	0	-	
	8-24	6890	110	757.9	
	Total		170	1030.9	0.089
**Animal 3**	0-4	7660	10	76.6	
	4-8	-	0	-	
	8-24	3700	6	22.2	
	Total		16	98.8	0.009
**Mean**				384	0.0332

In two dogs <20 ml of urine was collected and it is therefore likely that not all drug could be recovered in these animals. In the third dog, 170 ml of urine was collected and contains a higher fraction of compound (8.9%) than the other dogs and is therefore used for further studies. Source data are provided as Source Data file.

Supplementary Table 16. Plasma clearance

Species	Plasma clearance (ml/min/kg)				
	Predicted hepatic	Observed total	Observed renal	Observed total-renal = observed hepatic	Observed hepatic/ predicted hepatic
**Mouse**	7.0	13.4	n/d	<13.4	<1.9
**Rat**	13.3	21	7.7	13.5	1.0
**Dog**	11.1	11.5	1.0	10.5	0.95
**Human**	0.51				

Predicted hepatic clearance is based on the *in vitro* hepatic clearance data, Fu,p and B:P from Supplementary Table 11. The average of blood to plasma ratio in mouse, rat and human (0.95) was used in order to predict the hepatic clearance in dogs. The observed total clearance was derived from Supplementary Table 7-9 and the observed renal clearance was determined from Supplementary Table 15 and 16.

Supplementary Table 17. Mean fold induction of CYP1A2, CYP2B6 and CYP3A4 mRNA expression by MMV693183

Concentration (µM)	CYP1A2 mean fold induction			CYP2B6 mean fold induction			CYP3A4 mean fold induction		
	Donor 1	Donor 2	Donor 3	Donor 1	Donor 2	Donor 3	Donor 1	Donor 2	Donor 3
1.5	1.73	-	-	1.40	-	-	1.52**	-	-
5	1.48	1.11	1.38	1.75*	0.995	1.18	1.22	1.03	1.21
15	1.04	1.07	0.844	1.38	0.951	1.660	0.824	0.905	0.816
50	0.824	0.795	0.874	0.959	0.828	0.886	0.779	0.500**	1.26
150	0.704	0.613**	0.609	0.663	0.721*	0.727	1.19	0.477***	1.98
250	-	0.586**	0.436**	-	0.667**	0.582	-	0.665	2.86*
500	0.357**	0.496***	0.293***	0.495**	0.699**	0.325**	3.63***	1.49	3.16*

mRNA expression of CYP1A2, CYP2B6, and CYP3A4 was measured after a 72-hour incubation with MMV693183 in cryopreserved human hepatocytes from 3 donors. The mean fold induction of CYP enzymes is shown. Statistical significance was determined using an unpaired two-tailed t-test; * $p<0.05$; ** $p<0.01$; *** $p<0.001$.

Supplementary Table 18. Human hepatocyte clearance

Hepatocyte donor	MMV693183 CLint (µL/min/million cells)	7-Ethoxycoumarin CLint (µL/min/million cells)	Tolbutamide Clint (µL/min/million cells)
HC1010	0.4	6.277	0.293
HC02	0.082	21.23	0.048
HC06	0.142	3.136	0.265
HC425	0.0695	1.194	N.D.

N.D. Not determined. Source data are provided as Source Data file.

Supplementary Table 19. Human predicted PK parameters

Parameter	Value
CL, total (ml/min/kg)	1.11
CL, hepatic (ml/min/kg)	0.51
CL, renal (ml/min/kg)	0.60
V1 (l/kg)	0.843
V2 (l/kg)	1.97
Vss (l/kg)	2.81
Half-life (h)	32.4

Supplementary Table 20. PK model estimates for the final PK model of MMV693183 from *in vivo* female NSG mice

PARAMETER	VALUE	RSE (%)	SHRINKAGE	COMMENT
Fabs0	1 (FIX)	-	-	Relative bioavailability (-)
CL	1.25	41.70	-	Apparent clearance (l/h)
Vc	2.25	18.90	-	Apparent central volume (l)
Q1	0.242	167	-	Apparent intercompartmental clearance (l/h)
Vp1	799	347	-	Apparent peripheral volume (l)
Q2	0.558	54.90	-	Apparent intercompartmental clearance to second peripheral compartment (L/hour)
Vp2	1.81	28.70	-	Apparent second peripheral volume (L)
Tk0	0.364	126	-	Absorption time (h)
Tlag1	0 (FIX)	-	-	Absorption lag time (h)
**Inter-individual variability**				
omega(Fabs0)	0 (FIX)	-	-	Normal
omega(CL)	0.354	18.90	3.30%	LogNormal
omega(Vc)	0.195	56.70	57%	LogNormal
omega(Q1)	0.3 (FIX)	-	76%	LogNormal
omega(Vp1)	0.3 (FIX)	-	90%	LogNormal
omega(Q2)	1.59	18.90	6.10%	LogNormal
omega(Vp2)	0.343	36.50	38%	LogNormal
omega(Tk0)	0.1 (FIX)	-	94%	LogNormal
omega(Tlag1)	0 (FIX)	-	-	Normal
**Residual Variability**				
Additive error	0.00143	32.50	-	Compound concentration (ug/ml)
Proportional error	0.235	12	-	Fraction
Objective function	-265	-	-	-
AIC	-239			
BIC	-227			

Supplementary Table 21. Population model parameter estimates of the final PD model for MMV693183

PARAMETER	VALUE	RSE (%)	SHRINKAGE (%)	COMMENT
PLerr	0 (FIX)	-	-	Individual deviation from baseline parasitemia
GR	0.03 (FIX)	-	-	Net parasite growth rate (1/hour)
EMAX	0.51 (FIX)	-	-	Maximum clearance rate (1/hour)
EC50	0.0115	37%	-	Concentration achieving 50percent of maximum effect (µg/mL)
hill	2 (FIX)	-	-	Hill coefficient (.)
CLPara	0.0698	6.75%	-	Parasitemia clearance (1/hour)
**Inter-individual variability**				
omega(PLerr)	0.2 (FIX)	-	25%	Normal
omega(GR)	0 (FIX)	-	-	LogNormal
omega(EMAX)	0.1 (FIX)	-	24%	LogNormal
omega(EC50)	1.49	16.90%	8.10%	LogNormal
omega(hill)	0 (FIX)	-	-	Normal
omega(CLPara)	0.0412	197%	65%	LogNormal
**Parameter-Covariate relationships**				
beta_CLPara(STUDYCOV_1)	-0.931	6.66%	-	Study covariate on CLPara
**Residual Variability**				
error_ADD1	0.66	5.17	-	Log-transformed parasitemia (percent or 1/ml)
Objective function	1053	-	-	-
AIC	1061			
BIC	1065			

Supplementary Table 22. MIC and MPC_{90}

Parameter	Value
MIC	3.2 ng/ml
MPC_{90}	38.4 ng/ml
ED_{90}	4.4 mg/kg

Supplementary Table 23. Human dose estimation based on the log total parasite reduction

**Parameter Criteria**	**Total Clearance prediction method**	**Median (mg)**	**AUC plasma (mg*h/l)**	**C_{max} plasma (ng/ml)**
9 log total parasite reduction	*in vitro* hepatocytes	10	2.1	150
12 log total parasite reduction	*in vitro* hepatocytes	15	3.2	230
9 log total parasite reduction	Allometry	20	2.6	300
12 log total parasite reduction	Allometry	30	3.9	450

Supplementary Table 24. *In vitro* safety pharmacology of MMV693183

Toxicity	Test	Results
Off-target effects	Binding assay	All showed less than 25% inhibition/induction, except for GABA (non-selective) (25.2% induction) at 10 µM
	Enzyme and Uptake Assay	All showed less than 25% inhibition/induction, except for GABA transaminase (30.7% induction) and HDAC11 (36.9% inhibition) at 10 µM
	Patch clamp: hERG	IC_{50} > 10 µM
	Patch clamp: hNa_v 1.5	IC_{50} > 10 µM (Inhibition of 1.56% at 10 µM)
	Patch clamp: hK_V 1.5	IC_{50} > 10 µM (Inhibition of 2.15% at 10 µM)
	Patch clamp: hCa_V 1.2	IC_{50} > 10 µM (Inhibition of 24.20% at 10 µM)
Cytotoxicity	HepG2 viability	IC_{50} > 10 µM
	Primary human and rat hepatocytes viability	IC_{50} > 10 µM
Genotoxicity	AMES test	Non mutagenic in absence and presence of S-9 tested in 5 strains up to 5000 µg/plate. Differences in revertant numbers compared to control are depicted below<br><1.5-fold: TA102<br><2-fold: TA98, TA100<br><3-fold: TA1535, TA1537
	Micronucleus Screening Assay	No induction of micronuclei in human peripheral blood lymphocytes up to 2000 µg/ml in presence or absence of S-9 (rat liver metabolizing system)
Phototoxicity	UV test	No absorbance >290 nm

Source data are provided as Source Data file.

Supplementary Table 25. Liver weight at the end of the MTD1, PK and DRF studies

Study Type	Dose (mg/kg/day)	Liver weight (g) Males	Liver weight (g) Females
MTD1	0	6.58 (n = 3)	4.78 (n = 3)
MTD1	60	7.73 (n = 3)	4.77 (n = 3)
MTD1	200	8.66 (n = 3)	6.00 (n = 3)
MTD1	600	11.22 (n = 3)	7.44 (n = 3)
PK	60	11.4290 (n = 18)	6.5972 (n = 18)
PK	200	13.5439 (n = 18)	8.4371 (n = 17)
DRF	0	8.7790 (n = 3)	5.9330 (n = 3)
DRF	10	9.7817 (n = 3)	6.6517 (n = 3)
DRF	30	10.7203 (n = 3)	6.4437 (n = 3)
DRF	30	9.7543 (n = 3)	7.2357 (n = 3)

Source data are provided as Source Data file.

Supplementary Table 26. MTD1 study toxicity findings

	Males			Females		
**Dose (mg/kg day)**<br>**Number of Animals per group**	**60**<br>**3**	**200**<br>**3**	**600**<br>**3**	**60**<br>**3**	**200**<br>**3**	**600**<br>**3**
**Hypertrophy, hepatocellular ***	**2**	**2**	**3**	**0**	**1**	**3**
Minimal	2	1	1	-	1	2
Mild	-	1	2	-	-	1
**Hyperplasia bile duct***	2	1	**3**	0	2	**3**
Minimal	2	-	3	-	2	2
Mild	-	1	**-**	-	-	1
**Vacuolation, hepatocellular***	**0**	**0**	**0**	**2**	**3**	**2**
Minimal	**-**	**-**	**-**	2	2	-
Mild	**-**	**-**	**-**	-	1	2

* total number of animals with this toxicity

Supplementary Table 27. Toxicity in PK study

	**Males**		**Females**	
**Dose (mg/kg day)**	**60**	**200**	**60**	**200**
**Number of Animals per group**	18	18	18	17
**Hypertrophy, centrilobular** *	**18**	**18**	**14**	**17**
Minimal	1	-	8	5
Mild	13	8	5	10
Moderate	4	10	1	2
**Necrosis, hepatocellular***	**1**	-	**1**	**1**
Minimal	-	-	1	-
Mild	-	-	-	-
Moderate	1	-	-	-
Marked	-	-	-	1
**Increased mitoses, hepatocellular***	-	-	**1**	**1**
Minimal	-	-	1	1
Mild	-	-	-	-
**Extramedullary hematopoiesis***	**10**	**14**	**9**	**13**
Minimal	10	14	9	13

* total number of animals with this toxicity

Supplementary Table 28. Toxicokinetic exposure data, AUC

Study Type	Dose (mg/ kg day)	AUC$_{(0 - t)}$ (hr*mg/mL)											
		Males						Females					
		Day 1	Day 3	Day 4	Day 5	Day 6	Day 7	Day 1	Day 3	Day 4	Day 5	Day 6	Day 7
DRF	10	6.71	-	-	-	-	4.97	5.94	-	-	-	-	5.7
DRF	30	20.6	-	-	-	-	7.91	19.5	-	-	-	-	22
MTD1	60	41.2	-	-	-	-	11.4*	88.9	-	-	-	-	69.7
PK	60	47.4	16.6	12.4	83.3	61.4	67.6	45.3	42.3	44	41.1	36.3	48.4
MTD1	200	345	-	-	-	-	46.2	333	-	-	-	-	54.9
PK	200	228	31.2	21.3	19.2	19.8	24.6	214	118	71.1	29	19.6	19.4
MTD1	600	1060	-	-	-	-	147	1080	-	-	-	-	168

*Measured in 1 rat. Source data are provided as Source Data file.

Supplementary Table 29. Toxicokinetic exposure data, C_{max}

Study Type	Dose (mg/kg day)	C_{max} (mg/mL)											
		Males						Females					
		Day 1	Day 3	Day 4	Day 5	Day 6	Day 7	Day 1	Day 3	Day 4	Day 5	Day 6	Day 7
DRF	10	3.25	-	-	-	-	2.94	3.93	-	-	-	-	3.58
DRF	30	9.32	-	-	-	-	4.51	13.3	-	-	-	-	15.3
MTD1	60	35.5	-	-	-	-	15.6	73.1	-	-	-	-	59.5
PK	60	23.5	8.74	5.89	5.43	3.29	3.93	27.1	25.4	25.5	23.6	22.9	24.2
MTD1	200	234	-	-	-	-	33.3	219	-	-	-	-	53.6
PK	200	76.6	19.7	13.4	14.7	10.8	18.3	76.8	69.1	51	25.9	17	14.2
MTD1	600	501	-	-	-	-	124	496	-	-	-	-	168

Source data are provided as Source Data file.

Supplementary Table 30. Details on animal experiments

Study	Animals	Gender/ total # animals	Age	Weight	Control group	Positive Control	Drug formulation	Doses (mg/kg/day)	Days of treatment	Route	Food
Single dose activity	NOD-SCID mice	F/18	6-8 weeks	20-22 gram	No drug	1x 50 mg/kg Chloroquine	Tween80-EtOH ((70/30-1:10 H_2O)	50	1	PO	*Ad libitum*
G6PD hemolysis	NOD-SCID mice	F/25*	8 weeks	19.6 gram average	PBS	12.5 mg/kg Primaquine in PBS	Tween80-EtOH ((70/30-1:10 H_2O)	10, 25, 50	4	PO	*Ad libitum*
PKPD modeling	NOD-SCID mice	F/6	14-24 weeks	25-28 gram	No drug	DHA	Tween80-EtOH ((70/30-1:10 H_2O)	10, 25, 50, 100	1	PO	*Ad libitum*
		F/11	7 months	22-28 gram	No drug	2x10 mg/kg Chloroquine	Tween80-EtOH ((70/30-1:10 H_2O)	1x2.5<br>1x5<br>2x2.5<br>2x5	3<br>1<br>3<br>3	PO	*Ad libitum*
		F/9	7 months	22-28 gram	No drug	2x10 mg/kg Chloroquine	Tween80-EtOH ((70/30-1:10 H_2O)	5, 10, 25	1	PO	*Ad libitum*
Pharmacokinetics mice	CD1 mice	M/6	6-8 weeks	31-32 gram	N/A	N/A	5% DMSO, 95% saline	3 or 30	1	IV or PO	*Ad libitum*‡
Pharmacokinetics rats	Sprague Dawley rats	M/6		250±50 gram	N/A	N/A	5% DMSO, 95% saline for IV/5% DMSO, 95% saline, 0.5% carboxy methyl	3 or 30	1	IV or PO	*Ad libitum*‡

							cellulose and 0.1% Tween-80 for PO				
Pharmacokinetics dogs	Beagle dog	M/6	2-3 years	8-11 kg	N/A	N/A	2% DMSO, 9% Solutol HS15, 89% HPbC at 10% in water	1 or 2	1	IV or PO	Overnight fasting before dosing
Seven-day MTD1 repeat dose study in rats	Wistar Han rats	M/10, F/10	6 weeks	126-194 gram	Elix water	N/A	Elix water	60, 200, 600	7	PO	*Ad libitum*
PK toxicity study	Wister Han rats	M/18, F/18	9 weeks	M/220-245, F/146-173	N/A	N/A	Purified water	60, 200	7	PO	*Ad libitum*
MTD2 toxicity study	Wister Han rats	F/9	9 weeks	F/169-231 gram	N/A	N/A	Purified water	300, 600, 1000	3	PO	*Ad libitum*
DRF toxicity study	Wister Han rats	M/12, F/12	9 weeks	M/261-288, F/173-216 gram	Purified water	N/A	Purified water	10, 30	8	PO	*Ad libitum*

* 1 mouse was excluded prior to engraftment injections due to small size and hunched appearance, 2 mice did not meet inclusion criteria (%HuRBCs > 60%). ‡: Food fasting 12 h before dosing to 2 h after dosing. Gender F: female; M: male. Food fasting: fasting 12 h before dosing to 2 h after dosing; IV: intravenous; PO: *per os*.

Supplementary Table 31: Experimental design of the toxicity studies

Study	Study ID	CRO	Dosing (mg/kg/day); days	*In vivo* endpoints	Clinical Pathology	Pathology endpoints
MTD1	20154166	CR*	60, 200, 600; 7days	Morbidity/mortality, clinical signs, BW, BWG and FC	Hematology and Serum clinical chemistry	Gross pathology, organ weights and histopathology
PK	20223355	CR	60, 200; 7 days	Morbidity/mortality, clinical signs, BW, BWG and FC	-	Gross pathology, organ weights and histopathology
MTD2	20223357 Phase 1	CR	300, 600, 1000; 3 days	Morbidity/mortality, clinical signs, BW, BWG and FC	Hematology and Serum clinical chemistry	-
DRF	20223357 Phase 2	CR	10, 30; 8 days	Morbidity/mortality, clinical signs, BW, BWG and FC	Hematology and Serum clinical chemistry	Gross pathology, organ weights and histopathology

CR*: Charles River, the Netherlands
CR: Charles River, France
BW: Body Weight, BWG: Body Weight Gain compared to initial body weight, FC: Food consumption

Supplementary Table 32: Experimental design of blood sampling for toxicokinetic exposure

Study	Study ID	Animal number (per timepoint)	Blood sampling	
			Post-dose days	Post-dose hours
MTD1	20154166	2 Females + 2 Males	1 and 7	0.25, 1, 4, 8 and 24
PK	20223355	3 Females + 3 Males	1, 3, 4, 5, 6 and 7	0.5, 1, 2, 4, 8 and 24
DRF	20223357 Phase 2	3 Females + 3 Males	1 and 7	0.25, 1, 4, 8 and 24

Supplementary Table 33. Primers

Primer name	Primer sequence
**Sequencing & integration PCR primers**	
Sequencing guide	GCTTGGGGAGATCCGCC
Sequencing/Ct-Int-ACS-F	GGAAGAGATATAGATGGTACTGC
Sequencing-ACS-R	CGTCCAGATATCCAATAGTATCC
Ct-Int-ACS11-F	CAACCTATACGAAATAAATTTGGAGG
Ct-Int-R	CTCCAGTGAAAAGTTCTTCTCC
3'-Int-F	TGTATCTATAGTTTCAAGTAGGACG
3'-Int-ACS-R	AGGAGGGTATGATTTATTTCTTAAATC
3'Int-ACS11-R	GGTACAAATATATGCATACGAGAAC
**ACS T648M point mutation primers**	
Guide-F	TATTGATTAATGCTCTTAAGGCTGT
Guide-R	aaacACAGCCTTAAGAGCATTAATc
Mutation-F	CAATTTTATATGGCTCCGACGGCATTAAGAG
Mutation-R	CTCTTAATGCCGTCGGAGCCATATAAAATTG
HR-F-EcoRI	tataaagaattcGGATCAACTGGTAAACCTAAAGG
HR-R-AatII	aatatagacgtcTCTGAACGCTCCATCTCC
**ACS-GFP primers**	
Ct-F-NotI	aattatgcggccgcCAGGGCACAGATTAGGAGC
Ct-R-MluI	aataatacgcgtTTTCTTAATTTCAATATGCTTTAACTTTTTTTTAC
3'UTR-F-EcoRV-SacII	aattatgatatcccgcggTATATATATACCTTGTACTTACATTCGC
3'UTR-R-BstZ17i	aatattgtatacGCGTGCTTTTTGATTTTTGCC
**ACS11-GFP primers**	
Ct-F-NotI	aataatgcggccgcTTTTCATCTCCCATATCAGAAGG
Ct-R-MluI	attattacgcgtCTTATTTCTTGATTTATAAAGATCATTTATTAC
3'UTR-F-EcoRV-SacII	aatattgatatcccgcggAAAAGGGGTGAAATTAAAACAGACC
3'UTR-R-BstZ17i	aataatgtatacGGCAAGAAAAATAAAGCAGAAATC

Restriction sites are underlined

Supplementary References

1. Schalkwijk J, Allman EL, Jansen PAM, de Vries LE, Verhoef JMJ, Jackowski S, et al. Antimalarial pantothenamide metabolites target acetyl-coenzyme A biosynthesis in Plasmodium falciparum. Sci Transl Med. 2019;11(510).

Source Data Supplementary Figures

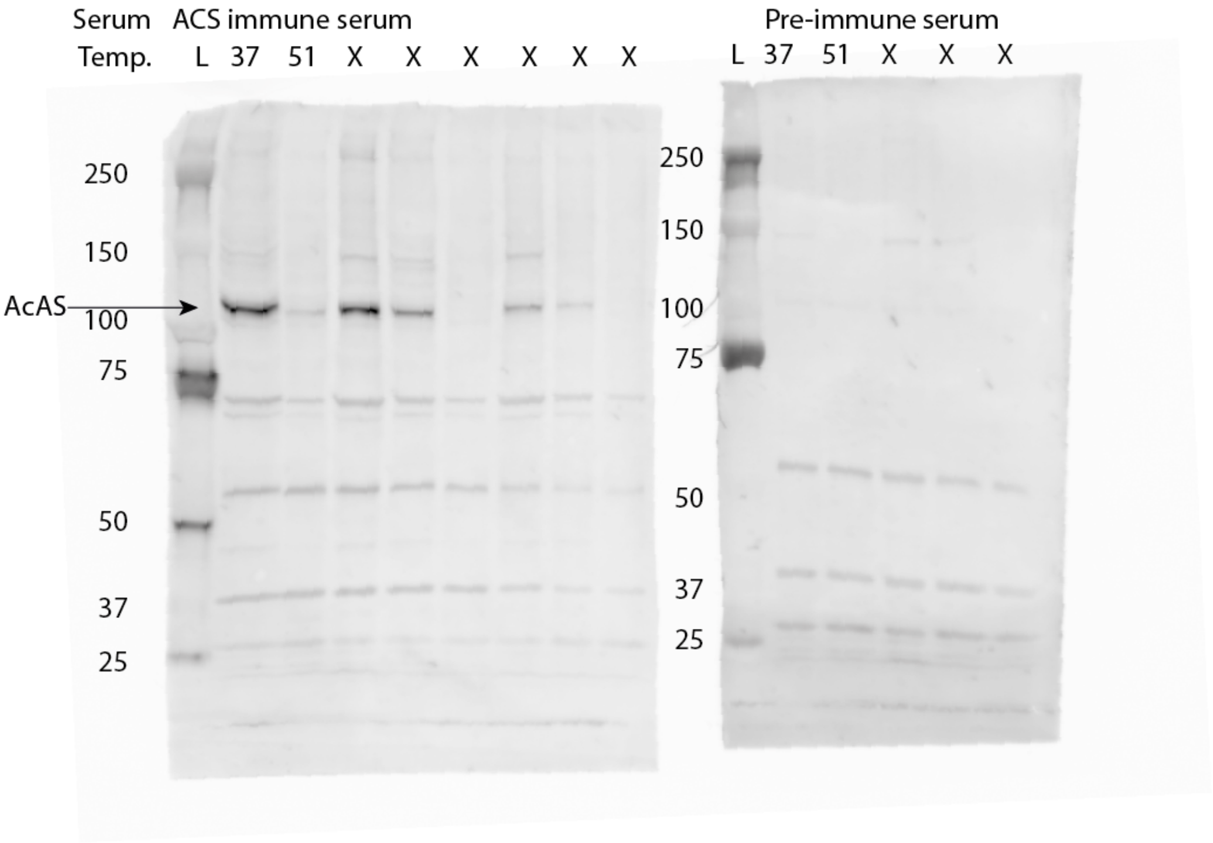

Original western blot of Supplementary Figure 11. L, ladder; X, lanes irrelevant to this study.

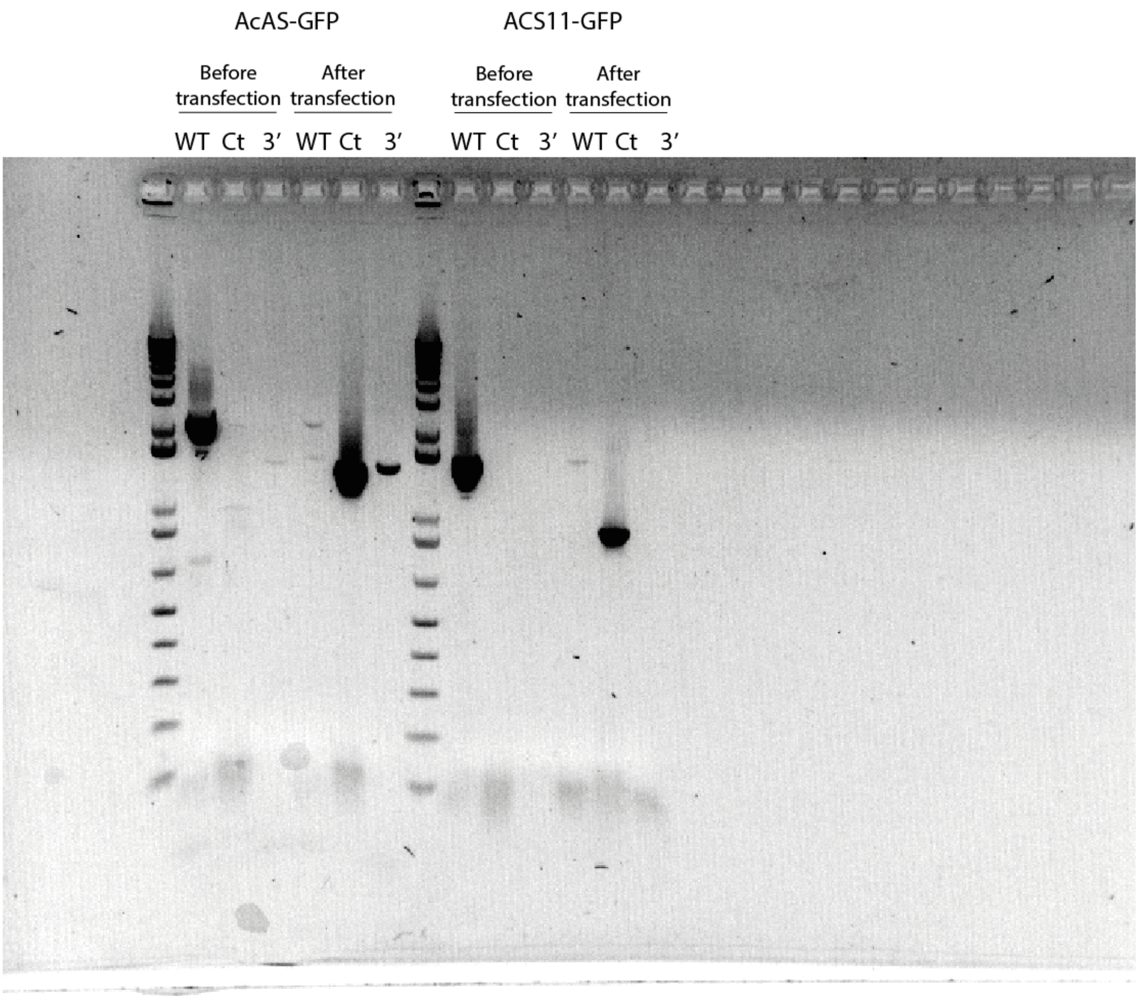

Original gel image of Supplementary Figure 12b.
