# Supplementary material for: Preclinical characterization and target validation of the antimalarial pantothenamide MMV693183
Source: Nat Commun. 2022 Apr 20;13:2158. doi: 10.1038/s41467-022-29688-5 (PMC9021288; doi:10.1038/s41467-022-29688-5)
Supplement: Supplementary file 3 — Reporting Summary [file 41467_2022_29688_MOESM3_ESM.pdf]

nature research

Corresponding author(s): Taco W.A. Kooij

Last updated by author(s): 24-02-2022

Reporting Summary

Nature Research wishes to improve the reproducibility of the work that we publish. This form provides structure for consistency and transparency in reporting. For further information on Nature Research policies, see our Editorial Policies and the Editorial Policy Checklist.

Statistics

For all statistical analyses, confirm that the following items are present in the figure legend, table legend, main text, or Methods section.

n/a	Confirmed	
☐	☒	The exact sample size (*n*) for each experimental group/condition, given as a discrete number and unit of measurement
☐	☒	A statement on whether measurements were taken from distinct samples or whether the same sample was measured repeatedly
☐	☒	The statistical test(s) used AND whether they are one- or two-sided<br>*Only common tests should be described solely by name; describe more complex techniques in the Methods section.*
☒	☐	A description of all covariates tested
☐	☒	A description of any assumptions or corrections, such as tests of normality and adjustment for multiple comparisons
☐	☒	A full description of the statistical parameters including central tendency (e.g. means) or other basic estimates (e.g. regression coefficient) AND variation (e.g. standard deviation) or associated estimates of uncertainty (e.g. confidence intervals)
☐	☒	For null hypothesis testing, the test statistic (e.g. F, t, r) with confidence intervals, effect sizes, degrees of freedom and P value noted<br>*Give P values as exact values whenever suitable.*
☒	☐	For Bayesian analysis, information on the choice of priors and Markov chain Monte Carlo settings
☒	☐	For hierarchical and complex designs, identification of the appropriate level for tests and full reporting of outcomes
☒	☐	Estimates of effect sizes (e.g. Cohen's *d*, Pearson's *r*), indicating how they were calculated

Our web collection on statistics for biologists contains articles on many of the points above.

Software and code

Policy information about availability of computer code

Data collection	GraphPad Prism (5.03, 9.2.0), Microsoft Excel (2016), CytExpert (2.3), BD C6 Plus, BD FACSDiva (8.0.1) were used to collect all data.
Data analysis	GraphPad Prism version 5.03, version 7 or version 9.2.0 were used to analyze the IC50s of all parasite growth curves. Phoenix WinNonlin version 6.4, version 1.4 were used for evaluation of the toxicokinetic parameters, PK parameters in dogs, rats and mice were calculated using Phoenix WinNonlin version 6.3. For PKPD modelling: Data preparation, exploration and model pre- and post-processing was performed using R (version 3.6.3) and R package IQRtools (version 1.2.1 IntiQuan GmbH), non-linear mixed effects (NLME) modeling was used to estimate the PK and PD parameters using Monolix (Lixoft version MLX2018R2). Fiji was used to process images and FlowJo (10.5.0, 10.7.1, 10.8.1) to analyze flow cytometry data and present the sorting strategy.

For manuscripts utilizing custom algorithms or software that are central to the research but not yet described in published literature, software must be made available to editors and reviewers. We strongly encourage code deposition in a community repository (e.g. GitHub). See the Nature Research guidelines for submitting code & software for further information.

Data

Policy information about availability of data

All manuscripts must include a data availability statement. This statement should provide the following information, where applicable:

- Accession codes, unique identifiers, or web links for publicly available datasets
- A list of figures that have associated raw data
- A description of any restrictions on data availability

The full metabolomics datasets are publicly available on the Metabolomics Workbench database under ST001985 (https://www.metabolomicsworkbench.org/data/DRCCMetadata.php?Mode=Study&StudyID=ST001985). The data associated with this study are presented in the paper, supplementary information and Source Data

file. Genetically engineered parasite lines that were generated for this study are available upon request under a material of transfer agreement. Source data are provided with this paper.

Field-specific reporting

Please select the one below that is the best fit for your research. If you are not sure, read the appropriate sections before making your selection.

☒ Life sciences ☐ Behavioural & social sciences ☐ Ecological, evolutionary & environmental sciences

For a reference copy of the document with all sections, see nature.com/documents/nr-reporting-summary-flat.pdf

Life sciences study design

All studies must disclose on these points even when the disclosure is negative.

Sample size	No sample size calculation was performed. Single dose activity screening to downselect the compounds was performed in as little as two humanized mice to reduce mouse use and costs. G6PD analysis was performed in groups of 5 mice which was previously shown to show the hemolytic toxicity of compounds (PMID: 24101478). All toxicology, PK and PD analyses were performed by commercial partners according to their protocols. For the majority of experiments, 2-4 biological replicates were performed. Typically, 2 replicates were performed for screening activities, as this would indicate whether there is an effect. A minimum of 3 replicates are performed for other experiments (exception mentioned below).
Data exclusions	Data from in vitro growth assays to test drug sensitivity/resistance in wild-type and genetically engineered parasites were excluded if the Z'value was below 0.5 or in the occassional situation of a pipetting or other technical error.
Replication	For the majority of experiments we performed 2-4 biological replicates, which were all successful. If applicable, technical replicates were performed, which were considered failed if control conditions were not presenting the expected results, if Z'values were below 0.5 for drug assays or if conditions of the assay, as described in the methods section, were not met. CETSA experiments performed with the CoA-PanAm and P-PanAm compounds were performed only once due to the very limited availability and extreme high costs of these compounds, however, this experiment supports the same final conclusion as other experiments that were replicated 2-4 times.
Randomization	Animals were randomly allocated to different treatment groups.<br>Charles River the Netherlands: Animals were assigned to groups by a computer-generated random algorithm according to body weights, with all animals within ± 20% of the sex mean. Males and females were randomized separately.<br>Charles River France: Animals were randomly assigned to groups at receipt.<br>G6PD hemolysis: the percentage of human RBCs in each mouse was calculated. Randomization was performed after calculations to ensure that there is no bias towards a particular treatment group having a higher or lower start percentage of human RBCs.<br>Single-dose activity study: non-computer aided, basic randomization was used to randomly allocate the animals to different groups.<br>Randomization of other experiments was not relevant as either mutant was compared to wild-type or the same batch of sample was used for all conditions.
Blinding	Blinding is not relevant to this study. All outcomes are objective, i.e. fluorescent signal for parasite growth, counting parasites, quantifying peak areas etc. and is therefore though to not bias our results.

Reporting for specific materials, systems and methods

We require information from authors about some types of materials, experimental systems and methods used in many studies. Here, indicate whether each material, system or method listed is relevant to your study. If you are not sure if a list item applies to your research, read the appropriate section before selecting a response.

Materials & experimental systems

n/a	Involved in the study	
☐	☒	Antibodies
☐	☒	Eukaryotic cell lines
☒	☐	Palaeontology and archaeology
☐	☒	Animals and other organisms
☐	☒	Human research participants
☒	☐	Clinical data
☒	☐	Dual use research of concern

Methods

n/a	Involved in the study	
☒	☐	ChIP-seq
☐	☒	Flow cytometry
☒	☐	MRI-based neuroimaging

Antibodies

Antibodies used	anti-Pfs25 monoclonal antibody: BEI Resources, cat no. MRA-315, clone 4B7. Conjugated to fluorophore Cy3 (GE Healthcare)<br>rabbit anti-HSP70: StressMarq, #SPC-186D, polyclonal, lot 1007<br>Alexa fluor 594 goat anti-rabbit IgG (H+L): Invitrogen, Cat no. #A11012, polyclonal<br>rabbit anti-ACS serum (Eurogentec)

GFP polyclonal antibody, chicken anti-GFP: Invitrogen, Cat no. A10262, lot no. 2156242
Goat anti-Chicken IgY (H+L) Secondary antibody, alexa fluor 488: Invitrogen/ThermoScientific, cat no. A11039, lot no. 1937504
FITC Rat monoclonal [YTH89.1] to Glycophorin A (Abcam. Cat No: ab28082; Lot No: GR3367206-1)
TER-119/Erythroid Cells Rat anti-Mouse, PE, Clone: TER-119 (Fisher. Cat No: 553673; Lot No: 0066166)
CD71 (Transferrin Receptor) Rat anti-Mouse, FITC, Clone: R17217 (RI7 217.1.4) (Fisher. Cat No: 11-0711-85; Lot No: 2043801)
Rat IgG2a kappa, FITC, Clone: eBR2a, Isotype Control (Fisher. Cat No: 11-4321-85; Lot No: 2086267)
Rat IgG2b kappa, PE, Clone: eB149/10H5, Isotype Control (Fisher. Cat No: 12-4031-82; Lot No: 1994100)

Validation

anti-Pfs25 monoclonal antibody: The murine hybridoma cell line, 4B7, was generated by the fusion of mouse myeloma cells with splenocytes from mice immunized twice with recombinant vaccinia virus expressing the 25-kDa gamete surface protein of Plasmodium falciparum (P. falciparum) (Pfs25), and boosted with whole P. falciparum gametes. This reagent was authenticated by the contributor and tested for mycoplasma. Each vial of MRA-315 contains approximately 0.5 mL of hybridoma cells (5 × 106 cells/mL) in cell culture medium supplemented with 10% dimethylsulfoxide (DMSO). Hybridoma 4B7 produces monoclonal antibody that specifically recognizes Pfs25 with no known cross reactivity. Barr, P. J., et al. "Recombinant Pfs25 Protein of Plasmodium falciparum Elicits Malaria Transmission-Blocking Immunity in Experimental Animals." J. Exp. Med. 174 (1991): 1203-1208. PubMed: 1940798.
rabbit anti-hsp70: Based on validation through cited publications. IFAs were performed on P. falciparum in liver stages in the following studies: Miyazaki et al., Front Cell Infect Microbiol, 2020; Tiburcio et al., mBio, 2019.
GFP polyclonal antibody: This Antibody was verified by Relative expression to ensure that the antibody binds to the antigen stated. Antibody specificity was demonstrated by detection of different targets fused to GFP tag in transiently transfected lysates tested. Relative detection of GFP tag was observed across different proteins fused with GFP in H3-GFP (Lane 3-5) and p65-GFP (Lane 6). GFP-variant, YFP is also being detected in His-p65-YFP lysate (Lane 7), using Anti-GFP Polyclonal Antibody (Product # A10262) in Western Blot. Immunofluorescent analysis of GFP Tag was performed using H3-GFP construct transfected in HEK-293E cells. The cells were fixed with 4% paraformaldehyde for 10 minutes, permeabilized with 0.1% Triton™ X-100 for 15 minutes and blocked with 2% BSA for 1 hour at room temperature. The cells were labeled with GFP Polyclonal Antibody (Product # A10262) at 1:100 dilution and Histone H3 Rabbit Polyclonal Antibody (Product # 711055) at 0.5 µg/mL in 0.1% BSA, incubated at 4 degree celsius overnight and then labeled with Goat anti-Chicken IgY (H+L) Secondary Antibody, Alexa Fluor 555 (Product # A-21437) and Goat anti-Rabbit IgG (H+L) Highly Cross-Adsorbed Secondary Antibody, Alexa Fluor Plus 647 (Product # A32733) respectively at a dilution of 1:2000 for 45 minutes at room temperature.
anti-ACS serum: Reactivity of serum was compared to pre-immune serum using an enzyme-linked immunosorbent assay. Briefly, plates were coated with 100 ng antigen per well and a dilution range of serum (pre-immune versus serum from final bleed) was added. Antibody binding was measured with a biotinylated goat anti-rabbit secondary antibody using the Vectastain ABC kit (Vector Labs). The previous steps were performed by Eurogentec. The ability of anti-ACS immune serum to detect ACS is studied in Figure S8 where parasite lysate was stained with immune serum or pre-immune serum on a Western blot, only showing a specific band in the immune serum. It is also tested in an IFA (Figure S10), showing that infected red blood cells showed a specific signal with immune serum, but only background with pre-immune serum.
FITC Rat monoclonal to glycophorin A: This antibody has been validated for flow cytometry only. The antibody has been used in the following three publications (PMID: 30588913, 24913163, 7688394)
TER-119/Erythroid Cells Rat anti-Mouse, PE: This is a monoclonal antibody that is routinely tested for flow cytometry applications. The antibody is verified in the following publication PMID: 10848813
CD71 rat anti-mouse, FITC: This antibody has been tested by flow cytometric analysis of mouse bone marrow cells. Since then it has been used for many (recent) publications.
Rat IgG2a kappa, FITC, Clone: eBR2a, Isotype Control: This rat IgG2a isotype control has been reported for use in immunocytochemistry, immunohistochemistry, and flow cytometric analysis. This rat IgG2a isotype control has been tested by flow cytometric analysis of mouse splenocytes. This can be used at the same concentration as the experimental antibody.
Rat IgG2b kappa, PE, Clone: eB149/10H5, Isotype Control: This monoclonal rat IgG2b kappa isotype control has been reported for use in flow cytometric analysis. Rat IgG2b Isotype Control has been tested by flow cytometric analysis of mouse splenocyte suspensions. It should be used at the same concentration as the experimental antibody.

Eukaryotic cell lines

Policy information about cell lines

Cell line source(s)

Dd2-B2, 3D7, NF54, HepG2. Experiments on Caco-2, and other cell lines than parasites are all performed by commercial partners according to their protocols.

Authentication

NF54: Radboudumc uses an authenticated master cell bank from which we regularly start new cultures; all genetic mutants generated in this background were made at the Radboudumc. Genetic mutants were verified by integration PCR and/or by Sanger Sequencing and shown in the supplementary information.
NF54-attb parasites were used for the conditional knockdown parasite lines. This parasite line has been generated and published previously (Adjalley et al. 2011). Integration of the conditional knockdown constructs was verified by western blots by showing protein of the expected molecular weight in presence of aTc, but not in absence of aTc, as well as by luminescence-based growth assays.
NF54 for the DGFA: NF54 was provided and genotyped by Leiden University Medical Centre.
For humanized mice experiments, parasites generated in Angulo-Barturen et al., Plos One, 2011 were used.
For the PRR assay, 3D7 from BEI resources were used.
For the metabolomics assays, 3D7 was used and obtained from the Malaria Research and Reference Reagent Resource (MR4) Center.
Dd2-B2: Dd2 parasites were obtained from T. Wellems (NIAID, NIH). Dd2-B2 is a genetically homogenous line that was cloned from Dd2 by limiting dilution in the Fidock lab (PMID: 28808258). P. falciparum parasite lines were authenticated by whole-genome sequencing and comparison to known genome sequence data.
HepG2: cell line was obtained through ATCC who did the authentication.

Mycoplasma contamination

Parasites that were used for metabolomics analyses were weekly tested for Mycoplasma, and cultures were always tested negative.

	The parental NF54-attb line was tested negative for mycoplasma before integrating the conditional knockdown construct. The NF54 parasite lines used for the DGFA was intermittently tested in the lab for mycoplasma and no contaminated cultures have been found in the last 5 years. Mycoplasma contamination was not assessed for other experiments.<br>Dd2-B2: Parasite lines were screened by PCR for Mycoplasma every 3–6 months and found to be negative.<br>NF54 for compound screening were tested for mycoplasma every year and found to be negative.<br>HepG2: Cell lines are tested for mycoplasma every year and found to be negative.
Commonly misidentified lines<br>(See ICLAC register)	No commonly misidentified lines were used.

Animals and other organisms

Policy information about studies involving animals; ARRIVE guidelines recommended for reporting animal research

Laboratory animals	All information on animals used in this study including species, strain, sex and age are reported in Supplementary Table 30 and housing conditions are mentioned in the methods section.
Wild animals	No wild animals were used in this study.
Field-collected samples	No field-collected samples were used in this study.
Ethics oversight	Animal experiments performed at The Art of Discovery (TAD) were approved by The Art of Discovery Institutional Animal Care and Use Committee (TAD-IACUC). This committee is certified by the Biscay County Government (Bizkaiko Foru Aldundia, Basque Country, Spain) to evaluate animal research projects from Spanish institutions according to point 43.3 from Royal Decree 53/2013, from the 1st of February (BOE-A-2013-1337). All experiments were carried out in accordance with European Directive 2010/63/E.<br>The animal experiments carried out at the Swiss Tropical and Public Health Institute (Basel, Switzerland) were adhering to local and national regulations of laboratory animal welfare in Switzerland (awarded permission no. 2303). Protocols are regularly reviewed and revised following approval by the local authority (Veterinäramt Basel Stadt).<br>Aptuit is committed to the highest standards of animal welfare and is subject to legislation under the Italian Legislative Decree No. 26/2014 and European Directive No. 2010/63/UE. Animal facilities are authorized by the Italian Ministry of Health with authorization n. 23/2017-UT issued on 29th November 2017 according to art. 20 of Legislative Decree No. 26/2014. Furthermore, general procedures for animal care and housing are in accordance with the Association for Assessment and Accreditation of Laboratory Animal Care (AAALAC) recommendations.<br>Animal procedures to determine the hemolytic toxicity were approved by the University of Colorado Anschutz Medical Campus Institutional Animal Care and Use Committee.<br>All animal studies had the approval of the Institutional Animal Ethics Committee (IAEC) of TCG Lifesciences Pvt. Ltd and were conducted in accordance with the guidelines of the Committee for the Purpose of Control and Supervision of Experiments on Animals (CPCSEA), Government of India.<br>The seven-day repeat dose study in rats was reviewed and agreed by the Animal Welfare Body of Charles River Laboratories Den Bosch B.V. within the project license AVD2360020172866 approved by the Central Authority for Scientific Procedures on Animals (CCD) as required by the Dutch Act on Animal Experimentation (December 2014).<br>Rat toxicity studies were performed at Charles River Laboratories (France) in accordance with the ICH S5(R2) guideline requirements and the respective Institutional Animal Care and Use Committees for care and treatment of laboratory animals. All animals were housed under standard laboratory conditions that have been approved by the respective Institutional Animal Care and Use Committees for care and treatment of laboratory animals.

Note that full information on the approval of the study protocol must also be provided in the manuscript.

Human research participants

Policy information about studies involving human research participants

Population characteristics	Uganda: 37.4% male, mean age: 5.275 years (median: 4, minimum: 0.5, maximum: 27, 25%percentile: 2, 75% percentile: 6), average parasitemia: 4.218% (median: 3.3%, minimum: 0.3, maximum: 20, 25% percentile: 2, 75% percentile: 5.5).<br>Brazil: 6 patients were male, 5 female; mean age was 37 years (minimum: 19; maximum: 58)<br>Cambodia: 86.7% male, mean age: 26.1 years (minimum: 10; maximum: 60)
Recruitment	Uganda: P. falciparum isolates were collected from patients aged 6 months or older presenting at the Tororo District Hospital (Tororo district) or Masafu General Hospital (Busia district) in eastern Uganda with clinical symptoms of malaria, a positive Giemsa-stained blood film for P falciparum, and no signs of severe disease. Patients reporting use of antimalarial treatment within the previous 30 days or with evidence of an infection with other Plasmodium species were excluded. No other exclusion criteria were applied. Written informed consent was obtained from all participants. Parents or guardians of children younger than 18 years provided written consent on their behalf; children aged 8–17 years provided assent. 2–5 mL of venous blood was collected in a heparin tube by a laboratory technician before the start of therapy. Participants were given artemether– lumefantrine, following national guidelines, after sample collection. The study was approved by the Makerere University Research and Ethics Committee, the UgandaNational Council for Science and Technology, and the University of California Committee on Human Research. 1) These samples are from infections with relatively high parasitemia (≥0.3%), and these results may not represent those from very low parasitemia infections, and 2) the samples are likely to be multiclonal, so the IC50 results are most likely to reflect averages of the various genotypes present in the samples.<br>Brazil: Patients recruited for the study were attended at the Malaria Control Center (CEPEM) in Porto Velho, Rondônia, Western Brazilian Amazon. Patients positive for single P. vivax or P. falciparum infection were invited to participate in the study. However, patients who showed severe symptoms of malaria (e.g., seizure, delirium, severe muscle pain, diarrhea) and needed prompt medical attention were not included in the study. They were referred to the hospital for tropical diseases in Porto Velho- CEMETRON. The exclusion of patients showing severe symptomatology could be considered a self-selection bias. For example, if the patient had a more virulent strain, the compounds would not be assessed against this more virulent

parasite strain.
Cambodia: The recruitment was done from P. falciparum mono-infected symptomatic individuals that seek treatment and accepted inclusion in the therapeutic efficacy studies performed between 2016 to 2019. Since the isolated parasites were adapted to in vitro conditions and maintained and tested under standardized conditions. Through this approach, the main confounding factors such as parasite stage, pre-exposure to drugs, immunity, were eliminated.

Ethics oversight

For collection of blood for ex vivo activity studies in Brazil and Uganda, all participants or their parents/guardians signed a written informed consent before blood collection. Patients were promptly treated for malaria after blood collection, following national guidelines. The ex vivo activity study in Brazil was approved by the Ethics Committee from the Centro de Pesquisa em Medicina Tropical - CEPEM-Rondônia (CAAE 61442416.7.0000.0011). The ex vivo activity study in Tororo, Uganda was approved by the Makerere University Research and Ethics Committee, the Uganda National Council for Science and Technology, and the University of California, San Francisco Committee on Human Research.
All isolates in the Cambodia study were collected during therapeutic efficacy studies (TES) upon protocol acceptance from the Cambodian National Ethical Committee (NECHR-077, NECHR-087, NECHR-092 & NECHR-099).

Note that full information on the approval of the study protocol must also be provided in the manuscript.

Flow Cytometry

Plots

Confirm that:

☒ The axis labels state the marker and fluorochrome used (e.g. CD4-FITC).

☒ The axis scales are clearly visible. Include numbers along axes only for bottom left plot of group (a 'group' is an analysis of identical markers).

☒ All plots are contour plots with outliers or pseudocolor plots.

☒ A numerical value for number of cells or percentage (with statistics) is provided.

Methodology

Sample preparation

G6PD hemolysis: Mice are assessed for percentage level of human red blood cell engraftment following the 14 days of intraperitoneal (IP) injections on days 0 (24 hours after last IP), 4, 5, and 7. To measure HuRBC levels, 3ul of whole blood is collected from each mouse via tail vein into Eppendorf tubes containing 100ul of heparin. For flow sample preparation, 1.5ul of each blood sample is transferred onto a 96-well plate with each well containing 100ul of a buffer solution (0.5% BSA/PBS). Samples are then stained with a 1:100 dilution of FITC anti-human glycophorin A antibody (Abcam. Cat No: ab28082) for HuRBC measurements and a mixed 1:400 dilution of PE anti-mouse TER119 (Fisher. Cat No: 553673) and 1:200 dilution of FITC anti-mouse CD71 (Fisher. Cat No: 11-0711-85) for murine reticulocyte measurements. Control samples are also stained and include: Unstained, FITC anti-glycophorin A, PE anti-TER119, and two FITC-PE isotype controls (Fisher. Cat No: 11-4321-85; Fisher. Cat No: 12-4031-82).
Resistant parasites: Flow cytometry-based analysis of Plasmodium falciparum parasitemias. Intra-erythrocytic parasites were cultured at a starting parasitemia of 0.3% and 1% hematocrit for 72 hr in the presence of a range of drug concentrations, with no-drug controls. Cells were then labeled with 1X SYBR Green I and 100 nM MitoTracker Deep Red (ThermoFisher) and parasitemias measured by flow cytometry using a BD Accuri C6 PLus flow cytometer.
Sorting endogenously-tagged parasites: Dilute parasite culture in media (20x) and sort with an ultra-pure sorting strategy

Instrument

G6PD hemolysis: Data is collected using the Beckman Coulter CytoFlex S Flow Cytometer (Model Number: B75442).
Resistant parasites: BD Accuri C6 Plus flow cytometer
Sorting endogenously-tagged parasites: BD FACSAriaII

Software

G6PD: Data were collected using CytExpert 2.3 and analyzed using FlowJo 10.7.1.
Resistant parasites: Data were collected using BD C6 Plus software and analyzed using FlowJo 10.5.0.
Sorting endogenously-tagged parasites: Data were collected using FACSDiva software version 8.0.1 and analyzed using FlowJo 10.8.1.

Cell population abundance

G6PD: no cells were sorted in this experiment
Resistant parasites: no cells were sorted in this experiment
Sorting of endogenously-tagged parasites: In both the AcAS-GFP and the ACS11-GFP parasites, the population was not pure as was observed after a PCR integration check (Figure S12b), yet enrichment of the GFP-tagged population allowed for more efficient imaging.

Gating strategy

The gating strategies of each experiment are described and indicated in figure S21, S23, and S25.
Gating strategy to determine hemolytic toxicity. Gating strategy for human RBCs: Blood samples were stained with anti-glycophorin A (FITC). RBCs are gated based on the FSC-A and SSC-A, subsequently human RBCs (HuRBCs) are selected based on their positive signal in the FITC channel in the RBC population (right). Gating strategy to quantify mouse reticulocytes: Blood samples were stained with anti-mouse TER119 (PE) and anti-mouse CD71 (FITC). From a RBC population, quadrants were made in the control samples stained with isotype controls. Mouse reticulocytes are presented in Q3.
Gating strategy for flow cytometry-based analysis of Plasmodium falciparum parasitemias. RBCs were gated based on the forward and side scatter. Live parasites were gated based on positive SYBR Green I and MitoTracker Deep Red signal in the RBC population.
Gating strategy to sort endogenously tagged parasites. An RBC population was selected based on the FSC-A and SSC-A plot. Subsequently, single cells 1 were selected from the SSC-H, SSC-W plot, followed by single cells 2 from the FSC-H, FSC-W plot. From this final population, GFP-positive cells were sorted.

☒ Tick this box to confirm that a figure exemplifying the gating strategy is provided in the Supplementary Information.
